# Impact of Geography and Climate on the Genetic Differentiation of the Subtropical Pine *Pinus yunnanensis*

**DOI:** 10.1371/journal.pone.0067345

**Published:** 2013-06-26

**Authors:** Baosheng Wang, Jian-Feng Mao, Wei Zhao, Xiao-Ru Wang

**Affiliations:** 1 Department of Ecology and Environmental Science, Umeå University, Umeå, Sweden; 2 National Engineering Laboratory for Forest Tree Breeding, Key Laboratory for Genetics and Breeding of Forest Trees and Ornamental Plants of Ministry of Education, Beijing Forestry University, Beijing, People’s Republic of China; 3 State Key Laboratory of Systematic and Evolutionary Botany, Institute of Botany, Chinese Academy of Sciences, Beijing, People’s Republic of China; Wuhan Botanical Garden, Chinese Academy of Sciences, China

## Abstract

Southwest China is a biodiversity hotspot characterized by complex topography, heterogeneous regional climates and rich flora. The processes and driving factors underlying this hotspot remain to be explicitly tested across taxa to gain a general understanding of the evolution of biodiversity and speciation in the region. In this study, we examined the role played by historically neutral processes, geography and environment in producing the current genetic diversity of the subtropical pine *Pinus yunnanensis.* We used genetic and ecological methods to investigate the patterns of genetic differentiation and ecological niche divergence across the distribution range of this species. We found both continuous genetic differentiation over the majority of its range, and discrete isolated local clusters. The discrete differentiation between two genetic groups in the west and east peripheries is consistent with niche divergence and geographical isolation of these groups. In the central area of the species’ range, population structure was shaped mainly by neutral processes and geography rather than by ecological selection. These results show that geographical and environmental factors together created stronger and more discrete genetic differentiation than isolation by distance alone, and illustrate the importance of ecological factors in forming or maintaining genetic divergence across a complex landscape. Our findings differ from other phylogenetic studies that identified the historical drainage system in the region as the primary factor shaping population structure, and highlight the heterogeneous contributions that geography and environment have made to genetic diversity among taxa in southwest China.

## Introduction

Genetic differentiation is strongly influenced by neutral processes and ecological selection. Population divergence engendered by geographical isolation as a consequence of topographical change and recent climatic oscillations are well documented in phylogeographic studies [1], [2]. In these situations, physical distance and geographical barriers are major factors limiting gene flow, and populations diverge via genetic drift. In contrast, under ecological selection, migration may occur between populations located in close proximity but adapted to distinct niches. However, the fitness of immigrants or hybrids may be less than that of an existing population in a given environment, and this will limit the potential for genetic exchange [3]. Thus, niche divergence and local adaptation may produce or maintain genetic divergence even if physical barriers to dispersal eventually disappear [4], [5], [6]. Determining the role of environmental factors in causing genetic differentiation and assessing their importance relative to that of historical isolation, have been challenging [7]. In recent years, the combination of informative molecular markers, spatial statistics and high resolution geographic information system (GIS) data has made it more feasible to carry out explicit evaluation of environmental influences on the distribution of genetic variation [8], [9], [10]. This approach offers the opportunity of assessing how specific landscape and environmental features have shaped gene flow between populations and the extent of local adaptation [11]. Correlation between environmental and genetic gradients can often provide initial evidence of the impact of natural selection and local adaptation [12], [13], [14]. Such information is important for understanding the neutral and selective processes driving divergence and, ultimately, speciation.

Southwest (SW) China is a biodiversity hotspot characterized by complex topography, heterogeneous regional climates and rich flora [15], [16]. In particular, Yunnan Province has a climate and ecology distinct from those of the majority of the Eurasian continent, in that much of the region has been free from glacial advances and retreats, creating a region with high biodiversity that has been maintained for millions of years [17], [18], [19]. Thus, local adaptation and ecological divergence have potentially had sufficient time to influence the pattern of genetic differentiation in many local species. For this reason, the area is particularly attractive for studies on the roles played by macro- and micro-evolutionary processes in the evolution of biodiversity and speciation. The topography of SW China is characterized by a number of large valley systems, e.g. those of the Jinsha (Upper Yangtze), Mekong and Salween Rivers. These deep valleys, together with the high mountains surrounding them, have been identified as strong geographic barriers to dispersal, which have defined the phylogeography of regional flora [20], [21]. The geometry and evolution of fluvial systems in this region have been affected to a great extent by tectonic changes in the Tibetan Plateau. During the most recent episodes of uplift of the eastern Tibetan Plateau, which occurred in the Late Miocene-Pliocene, major river drainage systems in SW China were reorganized and reinforced [22]. Species in this region responded uniquely to these landscape changes. In a number of conifers, herbs and shrubs, phylogeographic studies have revealed major landscape effects in which the current mountain and valley systems have acted as natural dispersal barriers [20], [23], [24], while in some other plants, freshwater fishes and amphibian species, the spatial genetic structure was found to reflect the historical geography of the region rather than the current geography [25], [26], [27], [28], [29], [30]. These phylogeographic analyses to date have concentrated primarily on the effects of neutral processes on the pattern of genetic variation, and the roles of environmental adaptation and ecology-driven genetic divergence have seldom been examined.

*Pinus yunnanensis* is a subtropical pine endemic to SW China, which has a continuous distribution in the Yunnan-Guizhou region at elevations ranging from 700–3000 m above sea level across all the major river valleys [31], [32]. Climatic conditions vary between regions divided by the mountain chains, and pronounced morphological variations in this pine have been recorded across its range [33], [34], [35], [36]. It has hybridized with another Asian pine, *Pinus tabuliformis*, generating a homoploid hybrid, *Pinus densata* [37], [38], [39], [40]. Mitochondrial (mt) and chloroplast (cp) DNA markers have been used to investigate the direction of hybridization and extent of introgression among these three species [39], [41]. Moderate levels of total mtDNA and cpDNA diversity were detected in *P*. *yunnanensis*, of which 45% and 4%, respectively, resided between populations [39]. In most of these early investigations, *P. yunnanensis* was used as a parental reference to characterize the hybrid nature of *P. densata* and therefore only relevant representative populations were sampled and analysed. The western and south-eastern marginal populations of *P. yunnanensis*, which have distinct ecological and morphological characters, were inadequately represented or not sampled at all in the previous mt- and cpDNA analyses. The species-wide pattern of genetic diversity in *P*. *yunnanensis* therefore remains unknown. Moreover, the ecological and phylogeographic processes responsible for the current population structure of *P*. *yunnanensis* have not been explicitly addressed. In this study, we sampled 16 populations throughout the range of *P. yunnanensis* to cover all ecological habitats, especially those on the western and south-eastern peripheries. Both mtDNA and cpDNA variations were analyzed, together with environmental data, in order to assess the influence of ecological and historical factors on genetic divergence in *P. yunnanensis*. We addressed the following questions: How is genetic diversity distributed geographically in *P. yunnanensis*, and does the observed genetic pattern reflect the modern or the historical geography? What is the extent of environmental heterogeneity within the species’ range, and could ecological factors have promoted genetic differentiation in this pine?

## Materials and Methods

### Ethics Statement

Here we state that the sampling of *P. yunnanensis* populations used in our study did not require any specific permission from any authority as it is a dominant forest species in SW China. Thus this study does not involve endangered or protected species.

### Population Sampling, Sequencing and Genotyping

**Figure 1:**
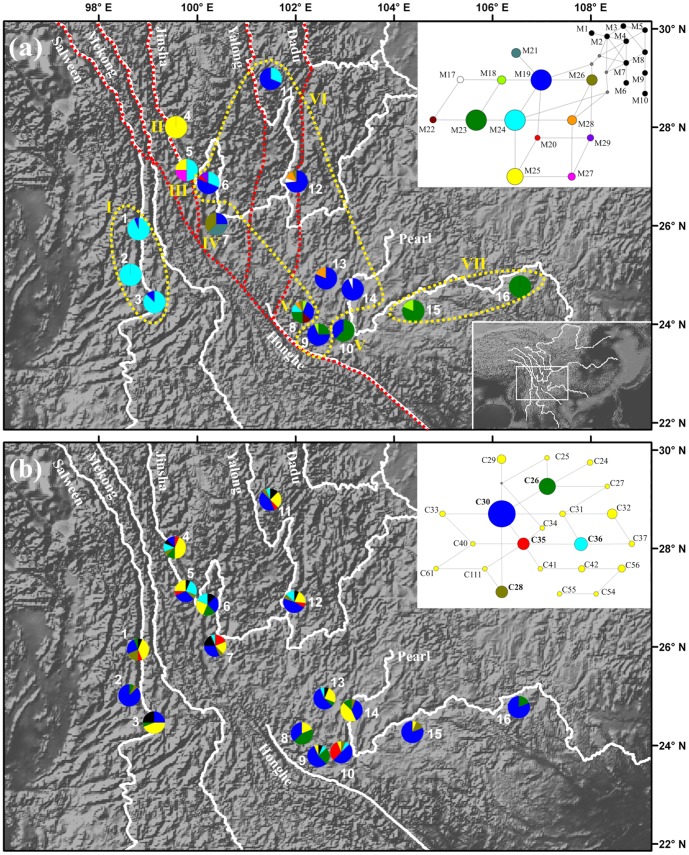
Mitotype (a) and chlorotype (b) composition of the 16 populations of *Pinus yunnanensis*. (a) Pie charts show the proportions of mitotypes in each population. Seven groups (I–VII) defined by mtDNA SAMOVA are shown. The current major rivers in Southwest China are illustrated in white, and the dashed red lines indicate the paleo-drainage routes before the major river reorganization (adapted from Clack et al. [22]). In the mtDNA network, each link represents one mutation step. Circle size is proportional to the frequency of mitotypes over all populations. Mitotype nomenclature follows that in Wang et al. [39]. M1–M10 occurred exclusively in *P. tabuliformis* and are used as an outgroup in this study. M17–M29 were detected in *P. yunnanensis* and are colored individually. (b) Pie charts show the proportions of chlorotypes in each population; singletons are grouped and shown in black. Relationships among 22 common chlorotypes are shown in the network, in which each link represents one mutation step. Circle size is proportional to the frequency of mitotypes over all populations. Chlorotype nomenclature follows that in Wang et al. [39]. The five most common chlorotypes (frequency >5%; C26, C28, C30, C35 and C36) are indicated in bold, and colored green, brown, dark blue, red and light blue, respectively.

We sampled 255 individuals from 16 populations throughout the range of *P. yunnanensis*. The distribution of these populations is illustrated in Fig. 1. The name, location, and sample size of each population are listed in Table 1.

**Table 1:** Geographic locations, sample sizes (*N*), number of haplotypes (*n*_h_), and genetic diversity (*H*_e_) of the 16 *Pinus yunnanensis* populations included in this study.

		Longitude	Latitude	Altitude	mtDNA			cpDNA			Population code
Population		(E)	(N)	(m)	*N*	*n*_h_	*H*_e_	*N*	*n*_h_	*H*_e_	in Wang et al. [39]
1	Gongshan	98°49'	25°58'	1616	16	2	0.125	16	9	0.900	49
2	Tengchong	98°39'	25°02'	1580	16	1	0	16	3	0.242	New
3	Baoshan	99°08'	24°28'	1897	16	2	0.233	16	11	0.933	50
4	Zhongdian 1	99°32'	28°09'	3048	16	1	0	16	9	0.917	45
5	Zhongdian 2	100°03'	27°11'	2009	16	3	0.667	16	8	0.858	46
6	Lijiang	100°13'	26°53'	2493	16	4	0.675	16	9	0.900	47
7	Binchuan	100°21'	25°58'	3141	16	3	0.700	16	9	0.883	48
8	Yuxi	102°09'	24°15'	1849	16	7	0.850	16	4	0.692	54
9	Shiping	102°29'	23°43'	1428	16	3	0.492	16	5	0.650	New
10	Jianshui	102°57'	23°50'	2084	16	2	0.500	16	5	0.683	New
11	Jiulong	101°30'	29°00'	3129	16	2	0.458	16	10	0.825	44
12	Miyi	102°01'	26°55'	2047	15	4	0.467	15	8	0.791	51
13	Kunming	102°37'	24°58'	2242	16	2	0.325	16	8	0.700	52
14	Yiliang	103°10'	24°43'	1846	16	2	0.125	16	8	0.850	53
15	Luoping	104°24'	24°17'	1643	16	2	0.325	16	3	0.342	New
16	Leye	106°34'	24°48'	1039	16	1	0	16	2	0.325	New
Total					255	13	0.773	255	39	0.778	

Eleven of the 16 populations (Nos. 1, 3–8 and 11–14) have been characterized for mtDNA and cpDNA variation in a previous study [39]. The data are based on three mtDNA segments (*nad*1 intron 2, *nad*4 intron 3 and *nad*5 intron 1) and five cpDNA microsatellite (cpSSR) loci (Pt45002, Pt71936, Pt87268, PCP1289 and PCP41131) [42], [43]. The other five populations (Nos. 2, 9–10 and 15–16) were collected *de novo* for this study. For these additional populations, composite seed samples were collected from more than 100 mature trees in each stand, and then 16 bulked seeds per population were used to grow small seedlings for genotyping. Total DNA was extracted using a Plant Genomic DNA Kit (Tiangen, Beijing, China) according to the manufacturer’s instructions. These new samples were characterized with the same set of mt and cp genetic markers used by Wang et al. [39]. For mtDNA, the purified PCR products were sequenced directly using an ABI 3730 automated sequencer (PE Applied Biosystems). For cpDNA, PCR products were resolved using a CEQ8000 capillary sequencer (Beckman-Coulter). Allele identification and genotyping were performed using CEQ8000 Fragment Analysis software (Beckman-Coulter).

### Haplotype Network Analysis

Mitochondrial DNA sequences were aligned using Clustal X 1.81 [44], and alignments were further refined manually. Unique mt sequences (mitotypes) for *nad*1, *nad*4, *nad*5 and the combination of these three mtDNA sequences were identified among the sampled individuals. Their relationships were then established by median-joining networks using Network v. 4.6.1.0 [45]. Ten mitotypes (M1–10) identified in a sister species, *P. tabuliformis* [39], were used as an outgroup in the mtDNA network. A complex 27-bp insertion/deletion region was found in each of the three mt segments when aligning the sequences between *P. yunnanensis* and *P. tabuliformis*. Following the approach of Wang et al. [39], we treated distinct sequence types in this 27-bp region as having arisen from different insertion events in order to obtain the most compact network.

For cpDNA data, size scores for the five cpSSR loci in each individual were combined into a 5-locus chloroplast haplotype (chlorotype). Relationships among the chlorotypes were reconstructed using the median-joining model implemented in Network. For simplicity, singletons were excluded from the network analysis.

### Genetic Diversity Analyses

All genetic diversity analyses were based on individual genotypes. For both mt- and cpDNA, the observed number of haplotypes and genetic diversity were calculated for each population and for the species. Genetic differentiation among populations and groups of populations were estimated by analysis of molecular variance (AMOVA) [46] with significance tests based on 10 000 permutations. These analyses were performed using Arlequin v. 3.0 [47]. The genetic divergence index (*D*) proposed by Jost [48] was also calculated using the software package SPADE (available at http://chao.stat.nthu.edu.tw/softwareCE.html). Jost’s *D* provides a measure of actual differentiation of haplotypic frequencies among populations that is mathematically independent of within-population diversity [48].

The population structure was analyzed by comparing two coefficients of population divergence for both mtDNA (*G*_ST_ and *N*_ST_) and cpSSR (*G*_ST_ and *R*_ST_). *G*_ST_ is based solely on allele frequencies, while *N*_ST_ (or *R*_ST_) takes into account similarities or relatedness among haplotypes. Thus, a significantly higher value for *N*_ST_ (or *R*_ST_) than for *G*_ST_ implies that closely related haplotypes occur geographically closer to each other than distantly related haplotypes, indicating significant phylogeographic structure. The program Permut & CpSSR v. 2.0 [49] was applied to compare *G*_ST_ *vs*. *N*_ST_ or *R*_ST_ values using 10 000 random permutations.

To further assess genetic structure in *P. yunnanensis*, the spatial variance in mitotype and chlorotype distributions was analyzed using SAMOVA 1.0 [50]. This program implements a simulated annealing approach to define groups of populations (*K*) that maximize the proportion of total divergence due to differences between groups of populations (*F*_CT_). For each mitotype and chlorotype dataset, *K* values ranging from 2 to 10 were tested to search for the *K* that gave the highest *F*_CT_. The significance of each *F*_CT_ was tested by simulating the annealing process 1000 times.

Finally, historical population expansion was assessed by mismatch distributions of both mt- and cpDNA data, using Arlequin. In this analysis, cpSSR data were coded in a binary format following the method described by Navascués et al. [51]. A total of 10 000 parametric bootstrap replicates was used to generate an expected distribution under a model of sudden demographic expansion [52], and to test the goodness-of-fit of the demographic model.

### Ecological Niche Modeling and Partial Mantel Test

We extracted ecological data from Mao & Wang [32] to perform ecological niche modeling for *P. yunnanensis*. These data consist of 148 geo-referenced occurrence records and 14 environmental variables (Table S1). The 148 occurrence points were filtered spatially such that only one point occurred within each 1 km^2^ grid cell (the maximum sampling resolution of our environmental data). We then used these locations for inclusion in GIS environmental layers. The 14 environmental variables were first examined for pairwise correlations within the distribution of *P. yunnanensis*. Highly correlated variables could result in over-fitting of niche models and should thus be removed. After evaluation, we retained eight variables with pairwise Pearson correlation coefficients *r*<0.70 for subsequent analyses (Table S1), to minimize over-fitting of niche models and improve the interpretability of niche axes in the multivariate analyses. All selected environmental layers were converted to the same resolution at a grid cell size of 30×30 arc-seconds (1 km^2^), and analyzed using the raster package (avaiable at http://raster.r-forge.r-project.org) in R and ArcGIS 9.2 (Environmental Systems Research Institute, Redlands, CA).

We performed a TwoStep clustering analysis in SPSS 13.0 (SPSS, Chicago) to quantitatively assess environmental heterogeneity within *P. yunnanensis*. This analysis estimates the number of ecotypic clusters within *P. yunnanensis* and their membership based on our 148 occurrence points and 8 environmental variables. Firstly, a sequential clustering approach was implemented to divide records into subclusters by constructing a modified cluster feature (CF) tree. The process scans records one by one and merges them into subclusters based on a distance defined by the log-likelihood decrease. Secondly, subclusters identified in step one were grouped into the desired number of clusters that maximize the Bayesian information criterion (BIC). For each ecotypic cluster pair, the relative contribution of environmental variables to their discrimination was evaluated by discriminant function analysis (DFA) using SPSS, and Wilks’ λ was used to test the null hypothesis that the two clusters have identical means for the specific variables.

We also performed principal components analysis (PCA) to further investigate ecological differentiation within *P. yunnanensis*. PCA was applied to scaled data for all eight environmental variables corresponding to 148 *P. yunnanensis* occurrence records, without a prior designation of ecotypic clusters. The relative contribution of each environmental parameter to the formation of niche spaces was then represented in a PCA distance biplot, and the magnitude and statistical significance of niche shifts among the occurrence clouds in the PCA graph were assessed using between-class inertia percentages and 99 Monte-Carlo randomization tests [53]. The PCA was performed and the PCA biplot generated using ade4 [54].

We then followed the procedure and parameter settings described in Mao & Wang [32] to construct the distribution range of each ecotypic cluster. Based on the 148 occurrence data points and 8 environmental variables, we simulated species distribution models (SDMs) via maximum entropy using Maxent 3.3.1 with default settings [55]. The predictive power of each model for the region where it was calibrated was evaluated, with 25% of the occurrence dataset being chosen at random and compared with the model output created with the remaining 75% of the present dataset. Ten thousand background points were sampled to construct a predicted range distribution for each *P. yunnanensis* cluster. Model accuracy was evaluated by assessing the area under the curve (AUC) of the receiver-operating characteristic (ROC) plot [56]. According to Swets’ scale [57], predictions are considered poor when AUC values are in the range 0.5–0.7, useful in the range 0.7–0.9, and good when greater than 0.9 (1 is perfect).

We also performed a niche-identity test to examine the null hypothesis that each pair of the ecotypic clusters is distributed in identical environmental space. This test compares the similarity of an ecotypic cluster’s actual niches to a distribution of niche similarities, obtained from pairs of pseudoniches based on randomly reshuffled occurrence points of the two clusters. The niche-identity test was performed in ENMTools [58] with 100 pseudoreplicates, and niche overlap between each pair of the ecotypic clusters was assessed by Schoener’s *D* [59] and Warren’s *I* [60] similarity index.

Finally, we applied a niche space-based multivariate test [61] to assess the possibility that the allopatrically-distributed ecotypic clusters occupy similar niches. This test compares background divergence (*d*_b_) with observed niche divergence (*d*_n_) in the PCA-reduced axes, with the null hypothesis *d*_b_ = *d*_n_ [61]. Niche divergence is supported if *d*_b_<*d*_n_ and the observed niche divergence itself (*d*_n_) is significant (according to a *t*-test), whereas niche conservatism is supported if *d*_b_>*d*_n_. For each climatic cluster, the eight environmental variables, longitude, latitude, and altitude were extracted from the occurrence points and from 1000 random background points within the background region of each ecotype using the packages dismo (available at http://cran.r-project.org/web/packages/dismo) and raster in R. The eight variables were reduced by PCA of the correlation matrix with the ade4 package. Correlations between the reduced PCA axes and the geographical variables (longitude, latitude and altitude) were examined by a nonparametric correlation test implemented in perm [62]. The background area for each cluster was delineated by SDMs from Maxent modeling at a baseline threshold obtained by minimizing the sum of sensitivity and specificity on the test data. In this study, *d*_n_ and *d*_b_ were computed as the differences between the mean scores of 75% random samples of the occurrence points of the two niches being compared (*d*_n_) and of the 1000 background points of the two compared background habitats (*d*_b_), in the reduced PCA axes. The distributions of *d*_b_ and *d*_n_ were generated with 1000 resamplings, and the mean of *d*_n_ was compared to the 95% confidence interval of *d*_b_ to determine its significance. The significance of the observed divergence between two compared niches was determined by a permutation *t*-test in perm.

To determine whether ecological factors explain genetic differentiation above and beyond differentiation due simply to isolation by distance (IBD), we performed partial Mantel tests on distances between populations. We compared matrices of pairwise genetic distance (*F*_ST_) vs. geographic distance and genetic distance (*F*_ST_) vs. ecological distance, controlling for ecological distance and geographic distance, respectively. Because the sample sites of all 16 populations were included in the 148 occurrence points for the species used in PCA, we estimated ecological distance by calculating the Euclidean distance between population pairs in a principal components space defined by the first two PC axes. Partial Mantel tests were performed with Arlequin, and 10 000 permutations were used in significance testing.

## Results

### Distribution of mtDNA and cpDNA Diversity

Sequences of the three selected mtDNA segments were obtained from 255 trees. When the three mtDNA segments were combined, a total of 13 mitotypes (M17– M29) were identified (Table S2). All of them have been reported by Wang et al. [39], and sequences of these mitotypes are available from GenBank accessions HM467712-HM467735. Network analysis showed that the 13 mitotypes were distinctly separated from those of *P. tabuliformis* (M1–M10), which was used as an outgroup, and all neighboring mitotypes differed by only one mutational step (Fig. 1a). A marked geographic pattern of mitotype distribution was observed in *P. yunnanensis*. The three most common mitotypes (M19, M23 and M24) were found in the central, south-eastern and western regions of the *P. yunnanensis* distribution, respectively. The other mitotypes were restricted to local populations at low frequencies, except for M25 which was fixed in population no. 4. In the case of the cp genome, a total of 39 chlorotypes (including 17 singletons) were detected over the five concatenated cpSSR loci (Table S2). After excluding the 17 singletons, network analysis of the 22 chlorotypes revealed a close relationship among them (Fig. 1b). The five most common chlorotypes (frequency >5%; C26, C28, C30, C35 and C36, which are shown in green, brown, dark blue, red and light blue, respectively in Fig. 1b) dominated all populations, with a total frequency of 72%, of which C30 (dark blue) contributed an overall frequency of 45%.

Total mtDNA diversity *H*_T_ (0.804) across all populations was much higher than the average within-population diversity *H*_S_ (0.371), resulting in strong between-population differentiation (*G*_ST_ = 0.538, *D* = 0.688). In contrast, the *H*_T_ value of 0.816 based on chlorotype variations was close to *H*_S_ (0.728), and both *G*_ST_ (0.108) and *D* (0.209) values were much lower than those for mtDNA (Table 2). AMOVA confirmed these findings, showing that 55.48% of the total diversity was due to population divergence for mtDNA, while only 6.94% in the case of the cpDNA divergence (Table 3). The levels of population differentiation observed in this study for both mt and cpDNA were higher than those previously reported (44.60% and 3.88%, respectively) for *P. yunnanensis* [39]. This increase is mainly caused by the inclusion of two new populations (Nos. 15 and 16), with distinct genetic compositions, from the south-eastern periphery. The contrasting pattern of population differentiation revealed by mtDNA and cpDNA loci in *P. yunnanensis* reflects the different modes of inheritance of the two cytoplasmic genomes. In the genus *Pinus*, variation in the mitochondrial genome represents the gene flow that is mediated by seed, while variation in the chloroplast genome represents gene flow attributable to both seed and pollen [63], [64].

**Table 2:** Average genetic diversity within populations (*H*_S_), total genetic diversity (*H*_T_), three coefficients of population divergence for mtDNA (*G*_ST_, *N*_ST_ and Jost’s *D*) and for cpDNA (*G*_ST_, *R*_ST_ and Jost’s *D* ), and mismatch distribution test for *Pinus yunnanensis*.

	No. of						Mismatch distribution			
	populations	*H*_S_ (SE)	*H*_T_ (SE)	*G*_ST_ (SE)	*N*_ST_ or *R*_ST_ (SE)	Jost’s *D*	*N*	τ	*P*_(SSD)_	Raggedeness index
mtDNA										
Species-wide	16	0.371 (0.068)	0.804 (0.036)	0.538 (0.091)	0.554 (0.091)	0.688 (0.016)	255	1.4	0.007	0.098**
Within groups†Group I	3	0.119 (0.067)	0.120 (0.063)	0.003 (0.062)	0.003 (0.062)	0 (0.016)	48	3.0	0.297	0.593
Group II	1	NC	NC	NC	NC	NC	NC	NC	NC	NC
Group III	1	NC	NC	NC	NC	NC	16	1.2	0.572	0.093
Group IV	1	NC	NC	NC	NC	NC	16	1.3	0.491	0.110
Group V	2	NC	NC	NC	NC	NC	32	1.7	0.111	0.124
Group VI	6	0.424 (0.075)	0.467 (0.077)	0.094 (NC)	0.066 (0.022)	0.076 (0.052)	95	1.8	0.518	0.140
Group VII	2	NC	NC	NC	NC	NC	32	3.0	0.184	0.452
Within ecotypes‡Py-eco1	3	0.119 (0.067)	0.120 (0.063)	0.003 (0.062)	0.003 (0.062)	0 (0.016)	48	3.0	0.299	0.593
Py-eco2	11	0.478 (0.076)	0.754 (0.074)	0.366 (0.110)	0.423 (0.116)	0.529 (0.038)	95	1.6	0.067	0.077
Py-eco3	2	NC	NC	NC	NC	NC	32	3.0	0.182	0.452
cpDNASpecies-wide	16	0.728 (0.058)	0.816 (0.053)	0.108 (0.030)	0.099 (0.026)	0.209 (0.049)	255	0.8	0.598	0.020
Within ecotypes‡Py-eco1	3	0.692 (0.225)	0.820 (0.173)	0.157 (0.228)	0.105 (0.262)	0.417 (0.140)	48	10.2	0.670	0.027
Py-eco2	11	0.812 (0.033)	0.870 (0.037)	0.067 (0.018)	0.132 (0.032)	0.138 (0.066)	95	2.0	0.689	0.030
Py-eco3	2	NC	NC	NC	NC	NC	32	3.0	0.617	0.202

SE, standard error; *N*, sample size; τ, expansion parameter; *P*_(SSD)_, SSD *P*-value; NC, not calculated due to low variation among populations;

** *P<*0.01;

† ,Grouping follows the division resulting from mtDNA SAMOVA;

‡ , Grouping follows the division resulting from TwoStep niche clustering analysis.

**Table 3:** Analysis of molecular variance (AMOVA) for mtDNA and cpDNA in *Pinus yunnanensis*.

	Source of variation	d.f.	SS	Variancecomponents	Percentageof variation	*F*-statistics
mtDNA						
	Among 16 populations	15	90.003	0.358	55.48**	*F*_ST_ = 0.55**
	Within populations	239	68.750	0.288	44.52	
	Total	254	158.753	0.646		
	Among 7 SAMOVA groups	6	85.411	0.413	57.77**	*F*_CT_ = 0.58**
	Among populations within groups	9	4.592	0.014	1.96**	*F*_SC_ = 0.05**
	Within populations	239	68.750	0.288	40.27**	*F*_ST_ = 0.60**
	Total	254	158.753	0.714		
	Among 3 ecotypes	2	40.262	0.267	34.41**	*F*_CT_ = 0.34**
	Among populations within ecotypes	13	49.741	0.222	28.58**	*F*_SC_ = 0.44**
	Within populations	239	68.750	0.288	37.01**	*F*_ST_ = 0.63**
	Total	254	158.753	0.777		
cpDNA	Among 16 populations	15	19.913	0.045	6.94**	*F*_ST_ = 0.07**
	within populations	239	144.950	0.606	93.06	
	Total	254	164.863	0.652		
	Among 3 ecotypes	2	5.265	0.025	3.72**	*F*_CT_ = 0.04*
	Among populations within ecotypes	13	14.648	0.033	4.92**	*F*_SC_ = 0.05**
	Within populations	239	144.950	0.606	91.36**	*F*_ST_ = 0.09**
	Total	254	164.863	0.664		

* *P*<0.05;

** *P*<0.01.

Comparisons of mtDNA *G*_ST_ *vs*. *N*_ST_ and cpDNA *G*_ST_ *vs*. *R*_ST_ indicated that *N*_ST_ and *R*_ST_ values were not significantly higher than *G*_ST_ values in *P. yunnanensis* (Table 2), suggesting a lack of phylogeographic structure in this species. However, mtDNA SAMOVA detected the presence of meaningful phylogeographic grouping, in which seven population groups (I–VII) were identified (Figs. 1 and S1). Groups I, VI and VII each spanned a large geographical area. Group I included three western populations (Nos. 1–3) dominated by mitotype M24, group VI included six central populations (Nos. 6, 9 and 11–14) dominated by M19, and group VII included two south-eastern populations (Nos. 15 and 16) in which M23 predominated. The other four groups (II–V) were each of restricted distribution in the central *P. yunnanensis* area. Groups II, III and IV were each represented by a single population, Nos. 4, 5 and 7, respectively, while group V included two populations, Nos. 8 and 10 (Fig. 1a). Group II was monomorphic for M25, while groups III–V each had multiple mitotypes in comparable proportions. SAMOVA of cpDNA variation failed to reveal any meaningful phylogeographic grouping (Fig. S1).

MtDNA-based mismatch distribution rejected a recent population expansion model at the species level (*P*_(SSD)_<0.01, Table 2), but supported it at group levels (*P*_(SSD)_>0.05). This analysis was not performed on group II because of its monomorphism. It was noticeable that the τ value of groups I and VII (3.0) was 2–3 times greater than that of the other groups (1.2–1.8). Based on the relationship τ = 2ut [52], the expansion time (t) is proportional to τ. If the mutation rate (u) is assumed to be constant within a species, the higher τ values of group I and VII would indicate that their expansion predated that of the other groups. A cpDNA-based mismatch distribution test indicated that a model postulating recent population expansion is supported at both the species and the ecotype level.

### Niche Differentiation across the Species’ Range

TwoStep clustering analysis grouped the 148 occurrence sites into three ecotypic clusters, Py-eco1, Py-eco2 and Py-eco3. These three clusters were geographically structured, and occupied the western, central and south-eastern ranges of *P. yunnanensis*, respectively (Fig. 3). DFA applied to all eight environmental variables supported the hypothesis that all ecotypic cluster pairs are significantly differentiated (Wilks’ λ, *P*<0.01, Table 4). Py-eco1 diverged from Py-eco2 mainly on the basis of wet day frequency (WET), while Py-eco3 diverged from both Py-eco1 and Py-eco2 on the basis of temperature variability (bio3).

**Figure 2:**
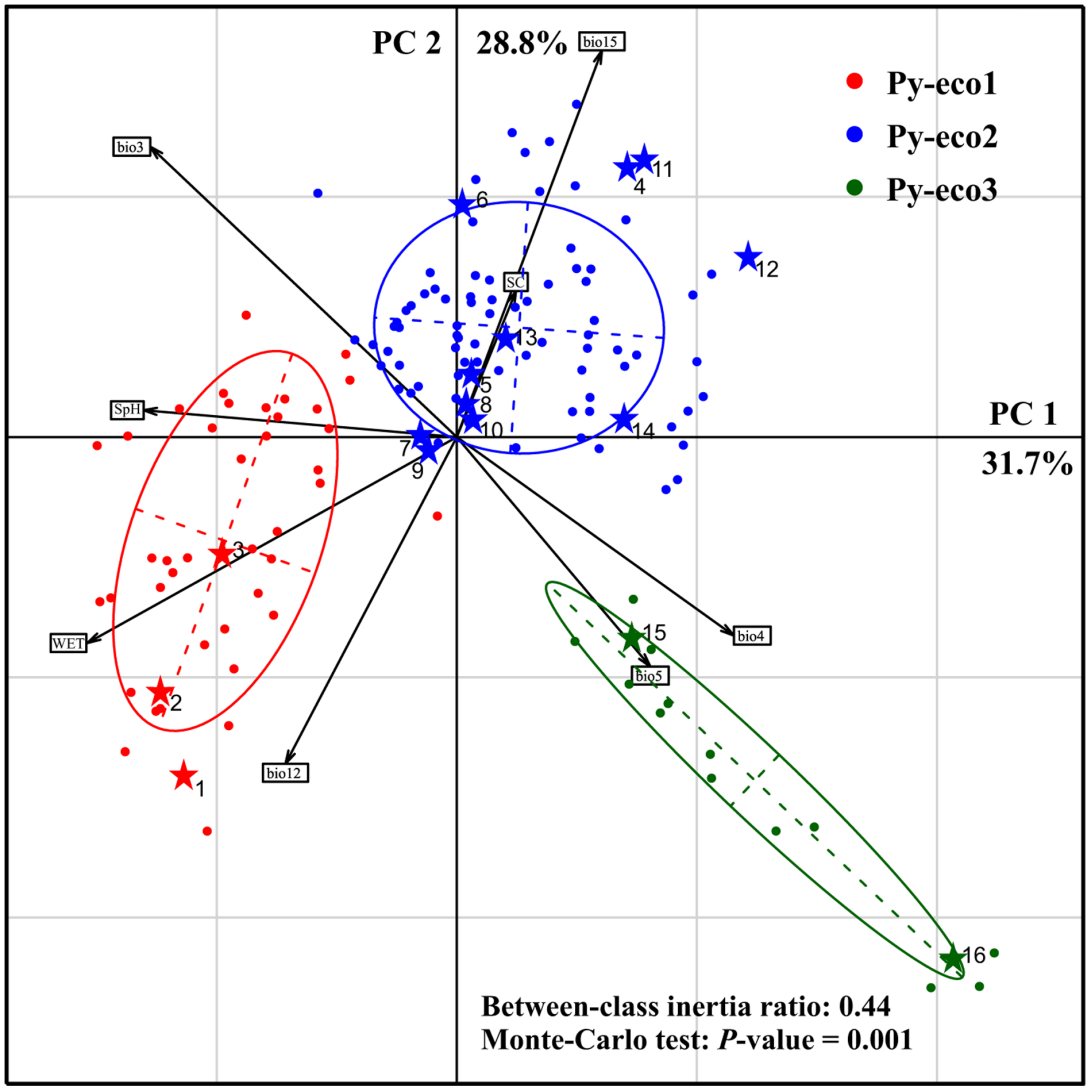
Principal components analysis distance biplot for the 148 *Pinus yunnanensis* occurrence sites based on eight environmental variables. Three occurrence clouds in the PCA graph are each outlined with a 1.5 inertia ellipse. The division of the 148 occurrence sites into three ecotypic clusters, Py-eco1, Py-eco2 and Py-eco3, by the TwoStep clustering analysis are shown in red, blue and green, respectively. The 16 populations sampled for genetic data analysis are each denoted by a star and numbered as in Table 1.

**Table 4:** The eight environmental variables (abbreviations in parentheses) used in this study, their contributions in discriminant function analysis (DFA) in pairwise comparisons of three ecotypes, and their similarities assessed based on Schoener’s *D* and Warren’s *I* index.

Environmental variables	Py-eco1 vs. Py-eco2	Py-eco1 vs. Py-eco3	Py-eco2 vs. Py-eco3
Isothermality (bio3)	0.17	**0.55**	**−0.83**
Temperature seasonality (bio4)	−0.06	**−0.27**	**0.41**
Maximum temperature of warmest month (bio5)	−0.06	−0.21	0.28
Annual precipitation (bio12)	**0.45**	0.01	**0.43**
Precipation seasonality (bio15)	**−0.44**	<0.01	−0.35
Soil organic carbon (SC)	−0.15	0.05	−0.27
Soil pH (SpH)	0.38	0.22	−0.19
Wet day frequency (WET)	**0.81**	**0.29**	0.02
Pairwise comparision			
DFA (Wilks’s λ)	0.21**	0.04**	0.21**
Schoener’s *D*	0.42	0.21	0.25
Warren’s *I*	0.73	0.37	0.45

Values corresponding to the three most significant variables are in boldface.

** *P*<0.01.

PCA of the eight environmental factors identified two components (with eigenvalues >1) that collectively explained 60.5% of the observed variation in the 148 occurrence records, accounting for 31.7% and 28.8% of the total variation, respectively (Fig. 2). The relative contributions of the different environmental variables to PC1 and PC2 are illustrated in the PCA distance biplot. PC1 is closely associated with temperature, soil type and seasonality (e.g. bio3, bio4, WET and SpH), while PC2 is associated mainly with precipitation (e.g. bio12 and bio15). The 148 occurrence sites were divided into three clearly separated environmental spaces in the Cartesian coordinates formed by the first two principal components. The niche centroids diverged strongly between the three clusters with a between-group inertia value of 0.44 (*P* = 0.001). This division was in good agreement with that produced by the TwoStep clustering analysis (Fig. 2). The PCA distance biplot shows that the three ecotypic clusters diverged from each other along both PC1 and PC2. According to the reduced dimensionality of the ecological spaces, Py-eco1 occupies a niche with a mild, moist and low-seasonality climate. Py-eco2 is more seasonal than Py-eco1, while Py-eco3 is characterized by a drier climate. Taken together, our results suggest that each of the climatic clusters identified here represents a niche with unique ecological characteristics.

**Figure 3:**
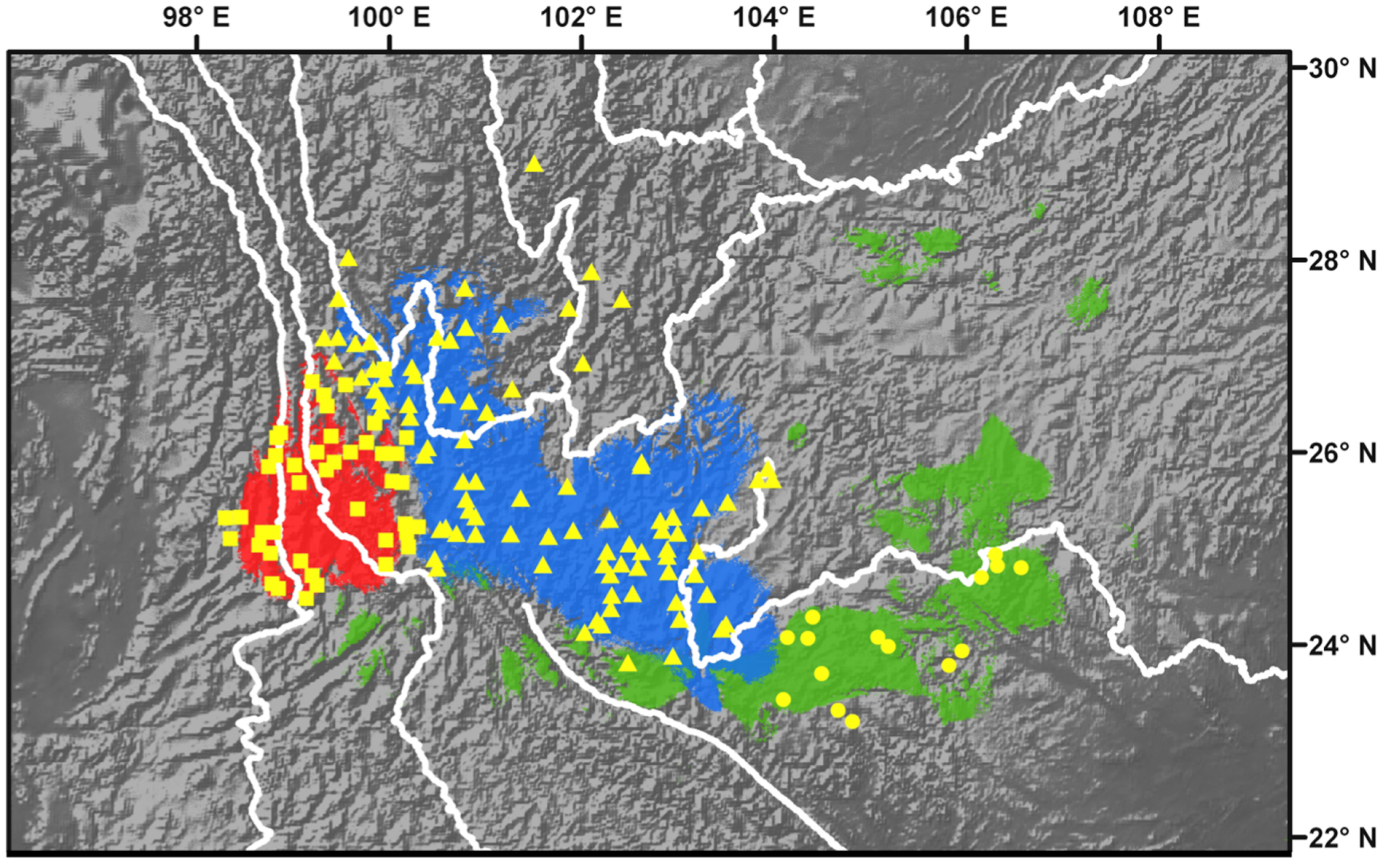
Predicted distribution for three *Pinus yunnanensis* ecotypes. The distribution ranges with probability of occurrence greater than 0.5, 0.5 and 0.6 for Py-eco1, Py-eco2 and Py-eco3 are shown in red, blue and green, respectively. Occurrence points used in the modeling are indicated by yellow squares, triangles and dots for Py-eco1, Py-eco2 and Py-eco3, respectively. The white lines show the current major rivers in southwest China.

Based on the occurrence records for each climatic cluster, we generated geographic distribution maps projecting the areas in which each cluster might occur (Fig. 3). The niche modeling accurately predicted the distribution of the three clusters identified by the TwoStep clustering analysis, with all training and test AUC values being greater than 0.99 (*P*<0.0001). The predicted distributions of the three clusters were generally consistent with the geographical ranges of their occurrence points, except that the projection for Py-eco3 extended beyond the observed distribution range of the species (Fig. 3). This inconsistency could be due to the limited numbers of occurrence points from the Py-eco3 region used in the modeling, which may have resulted in overestimation of the range of Py-eco3.

The niche similarity between Py-eco1 and Py-eco3 (Schoener’s *D* = 0.21 and Warren’s *I* = 0.37) was the lowest among the three niche pair comparisons (Table 4). A background test conducted by a multivariate method supported significant niche divergence between the three ecotypic clusters. Four axes were identified (each with an eigenvalue >1) that explained more than 85% of the total variation in each of the three pairwise comparisons (Table 5). In all pairwise tests, divergence was detected along all niche axes (i.e. *d*_n_>*d*_b_, and *d*_n_ is significant), except in the comparisons of PC1 for Py-eco1 vs. Py-eco3 and Py-eco2 vs. Py-eco3 (Table 5).

**Table 5:** Divergence on the independent niche axes between niche pairs.

	Py-eco1 vs. Py-eco2				Py-eco1 vs. Py-eco3				Py-eco2 vs. Py-eco3			
	PC1	PC2	PC3	PC4	PC1	PC2	PC3	PC4	PC1	PC2	PC3	PC4
*d*_n_	2.22** D	1.80** D	0.26** D	0.43** D	2.21** C	1.90** D	0.60** D	0.67** D	2.47** C	0.55** D	0.49** D	0.22** D
*d*_b_	1.99, 2.14	0.89–1.02	0.10–0.21	0–0.10	2.55–2.68	0.67–0.78	0–0.06	0.30–0.40	2.81–2.95	0.37–0.48	0–0.10	0.70–0.14
Top-loading variable	bio4, wet	bio5, sph	bio3, bio15	bio12, sc	bio3, bio15	wet	bio12	sc	bio3, bio15	bio4	wet	wet, sph, sc
% variance explained	38.89	19.02	14.63	13.46	42.88	15.79	15.26	12.69	48.62	15.32	13.16	8.13
Biological	Temperature,	Temperature,	Seasonality	Water,	Seasonality	Moisture	Water	Soil C	Seasonality	Temperature	Moisture	Moisture,
interpretation	Moisture	Soil PH		Soil C								soil PH & C
Correlation longitude	−0.36**	−0.62**	−0.26**	−0.17**	−0.91**	−0.23**	−0.11*	−0.05**	−0.85**	0.11**	0.38**	−0.09**
Correlation latitude	−0.72**	0.42**	0.25**	−0.01	−0.12**	0.25**	−0.39**	0.71**	0.09**	0.84**	−0.08**	0.15**
Correlation altitude	−0.52**	0.59**	0.44**	0.27**	0.81**	0.08**	−0.08**	0.39**	0.81**	0.27**	−0.15**	0.38**

Bold values indicate significant niche divergence (D) or conservatism (C) compared to a 95% null distribution (*d*_b_; *t*-test, ** for *P*<0.01). Significance of correlations between PC axes and geographical variables is indicated by * for *P*<0.05, and ** for *P*<0.01.

*d*_n_, observed niche divergence; *d*_b_, background divergence (95% null distribution).

### Effects of Environmental and Geographical Factors on Genetic Differentiation

The ecological niche clusters are broadly congruent with the grouping obtained by mtDNA SAMOVA. Py-eco1 and Py-eco3 correspond to SAMOVA group I and VII, respectively, while Py-eco2 covers populations from all other groups (II–VI) from the central distribution. Hierarchical AMOVA for mtDNA variation showed significant divergence between ecotypic clusters, since 34.41% of the variation occurred among ecotypes (Table 3). In contrast, genetic differentiation on the basis of cpDNA was low (3.72%) between ecotypic clusters (Table 3).

For mtDNA, partial Mantel tests across 16 populations detected low but significant correlations between genetic distance and geographic distance (*r*_gen-geo_ = 0.22, *P*<0.05; controlling for ecological distance) and between genetic distance and ecological distance (*r*_gen-eco_ = 0.28, *P*<0.05; controlling for geographic distance; Table 6). At the ecotype level, this test could be performed only for Py-eco2, due to the low level of polymorphism and limited number of populations in Py-eco1 and 3. In Py-eco2, population genetic distance correlated only with geographic distance (*r*_gen-geo_ = 0.18, *P*<0.05; *r*_gen-eco_ = 0.03, *P*>0.05). For the same reason, this test could be performed for only one (group VI) of the seven mtDNA SAMOVA groups. Within group VI, the genetic distance correlated with neither geographic nor ecological distance (*r*_gen-geo_ = −0.12, *P*>0.05; *r*_gen-eco_ = 0.20, *P*>0.05). For cpDNA, the genetic distance correlated with geographic distance only at the species level (*r*_gen-geo_ = 0.25, *P*<0.01; *r*_gen-eco_ = −0.06, *P*>0.05), and within Py-eco2 (*r*_gen-geo_ = 0.35, *P*<0.01; *r*_gen-eco_ = −0.25, *P*>0.05; Table 6). These results indicate that both geographic and environmental factors contributed to the pattern of mtDNA variation across the species as a whole, but only geographic distance affected cpDNA relatedness between populations.

**Table 6:** Correlation of genetic distance (*F*_ST_) with geographic and ecological distance (controlling for ecological and geographic distance, respectively) as measured by a partial Mantel test.

		Correlation of *F*_ST_ with geographic distance		Correlation of *F*_ST_ with ecological distance	
Spatial scale	No. of populations	*r*	*P*	*r*	*P*
mtDNA					
Species-wide	16	0.22	0.014	0.28	0.038
Group VI	6	−0.12	0.677	0.20	0.306
Py-eco2	11	0.18	0.039	0.03	0.397
cpDNA					
Species-wide	16	0.25	0.007	−0.06	0.629
Py-eco2	11	0.35	0.006	−0.25	0.927

## Discussion

### Phylogeography of *P. yunnanensis*

Located on the south-eastern margin of the Tibetan Plateau, SW China has undergone dramatic geomorphological changes during the most recent uplift of the plateau since the Late Miocene-Pliocene [65], [66], [67]. Reconstruction of the paleo-landscape of the region suggests that a large-scale dendritic drainage network formed on a regional low-relief landscape [65], [66], [67]. The initial drainage system was characterized by multiple southward-flowing rivers draining into the South China Sea through the ancient Honghe River (Fig. 1a). This landscape was destroyed by a series of river reversal and capture events and aggressive river incision in response to the uplift of the eastern plateau that was initiated between 13 and 9 million years ago (MYA) [65], [67]. The paleo-Mekong and Salween Rivers separated from the paleo-Honghe River, forming parallel rivers. The modern Mekong River drains southward into the South China Sea independently from the Honghe River, and the modern Salween River drains into the Indian Ocean. The paleo-Honghe River then split into two further systems: the northern branches, the Jinsha, Yalong and Dadu Rivers, connected to become the modern Jinsha River, which radically altered its southward course to an eastward one and now drains into the East China Sea (see [22] and Fig. 1a). The southern section of the paleo-Honghe River, which became disconnected from the upper streams, drains into the South China Sea following the course of the paleo-river. The southeastern margin of the Tibetan Plateau is now characterized by localized gorges, 2–3 km in depth, which major rivers have incised into the regionally elevated, low-relief, relict topography which represents the landscape that existed throughout the eastern margin prior to regional uplift [66], [67].

Molecular phylogeographic studies of endemic freshwater fishes and amphibian species in this region have suggested that the river rearrangements facilitated their genetic divergence, with estimates of divergence time falling between the Late Miocene and the Pleistocene [25], [26], [27], [28]. However, our understanding of the impact of landscape changes on the distribution, evolution and genetic structure of plant taxa of the region is limited (but see [29], [30]). *Pinus yunnanensis* is a dominant conifer of SW China with a continuous distribution, which probably extended beyond its extant range further into the north before the uplift of the eastern Tibetan Plateau [39]. We are interested to know whether the phylogeographic structure of *P. yunnanensis* has been strongly influenced by landscape changes in the past. Such studies are needed in order to understand the role played by habitat structure in the evolution of biodiversity in regional flora.

Analysis of mtDNA diversity revealed both discrete and continuous spatial structure in *P. yunnanensis*. MtDNA SAMOVA divided the species into 7 groups. Group I populations (Nos. 1–3) are located to the west of the paleo-Honghe River, and well separated from the other groups. This group is dominated by M24, a mitotype also detected in three northern populations (Nos. 5, 6 and 11) at relatively high frequencies. In addition, this mitotype was found in the ancient hybrid zone between *P. yunnanensis* and *P. tabuliformis* located allopatrically north of the current *P. yunnanensis* range [39], suggesting that historically M24 was widespread in the northern region. The sharing of M24 between group I and the northern populations indicates that there was probably a connection between these regions before the uplift of the eastern Tibetan Plateau. The low relief of the paleo-landscape would have facilitated regional population connectivity, and this pattern is still visible in the extant population structure due to the low mutation rate and non-recombinant nature of the mt genome and the long generation time of pine species. During the landscape changes that took place in the Late Miocene, M24 drifted to near fixation in the western periphery, and the region became isolated from seed exchange by the wide Salween and Mekong Rivers that function as barriers to seed dispersal. Similarly, group VII populations (Nos. 15 and 16), which represent the most south-easterly range of *P. yunnanensis*, are dominated by M23, a mitotype shared with three other southern populations (Nos. 8–10). Although the Pearl River separates group VII from the other populations, connectivity in this southern range is apparent, and M23 has drifted to a high frequency in the south-eastern periphery. It might be argued that the regional fixation of M24 and M23 could be due to introgression from neighboring species. This hypothesis seems unlikely because these two mitotypes were not detected in three other pines of the subgenus *Pinus* found in nearby regions, *Pinus massoniana*, *Pinus kesiya* and *Pinus merkusii* [39].

Spatial expansion can favor the fixation of low frequency alleles by drift in newly colonized areas [68], [69]. The mismatch distribution observed for mtDNA suggested that *P. yunnanensis* was in population equilibrium and had not undergone recent demographic expansion at the species level, but regional expansion was detected for all population groups. In addition, expansion in group I and VII seemed to have occurred earlier than that in other groups. This result suggests that groups I and VII were not established by recent colonization from the central area of the *P. yunnanensis* range. In this scenario, long-term isolation of group I and VII could help to reinforce population differentiation after colonization. Other factors, such as local adaptation during the period of isolation, may also have contributed to genetic divergence of groups I and VII; this issue will be discussed in the next section.

The central region of the *P. yunnanensis* range (groups IV–VI, population nos. 6–14) is characterized by the presence of the M19 mitotype at high frequencies. These populations are all located on the eastern side of the paleo-Honghe River but separated by the paleo-Jinsha, Yalong, and Dadu Rivers into parallel zones. This separation, however, does not seem to have impaired migration across the zones. After the reversal of the middle Jinsha River and the capture of its major tributaries by the East China Sea, the paleo-network became separated into disconnected northern and southern sections [22]. The midstream of the Jinsha River is deeply incised (>1000 m) into bedrock gorges, which could present an effective barrier to gene flow, especially that mediated by seed. The sharing of the M19 mitotype between these areas is not consistent with the modern landscape, but rather reflects the historically continuous distribution of populations along the southward paleo-Honghe drainages. The retention of ancient genetic structure in these central populations is probably attributable to the generally continuous distribution of the species, which reduces the effect of genetic drift.

Two populations (Nos. 4 and 5) in the north-west each had a unique mitotype composition and lacked the M19 mitotype which was found in the neighboring region. MtDNA SAMOVA identified each of these two populations as a distinct group (II and III, respectively). They were distributed along the paleo-Jinsha River, but separated from the other populations along this river by a sharp bend in the current river course. Geological analysis carried out by Clark et al. [22] suggests that the localized reversal of river segments at the capture points of the Yalong and Jinsha bends may be related to the effects of geo-activity on local structures rather than to have resulted from large-scale initiation of plateau uplift along the entire south-eastern Tibetan Plateau margin. Thus, the establishment of these two populations is likely to have been linked to the formation of the modern local topography. Because of their limited distribution, distinct mitotypes could have undergone rapid drift in these populations during range expansion that probably radiated from the neighboring group VI population. Upstream of the Jinsha, Mekong and Salween rivers, there is a region containing another closely related pine, *P. densata* [32]. The population-specific mitotypes (M25, M27) detected in populations Nos. 4 and 5 were not found in *P. densata* [39], a result which refutes the possibility of maternal introgression from *P. densata*, and further confirms that these two populations have been isolated from seed flow from nearby regions.

Taking all these results into consideration, we propose that the distribution of mtDNA variation in *P. yunnanensis* has been shaped by both the paleo-landscape and the formation of the modern regional topography. The connectivity between populations throughout the main range of the species reflects continuous distribution over a low relief paleo-landscape. This finding is similar to that of other phylogeographic studies of the region [25], [26], [27], [28], [29], [30], in that the modern landscape does not fully reflect the population structure, but it differs from other observations in that the distinct paleo-river shaped genetic structure seen in other river valley-limited taxa is not apparent in *P. yunnanensis*. Rather than showing that historical drainage systems played a major role in determining current intraspecific genetic structure, the observation of continuous genetic differentiation over the main range of *P. yunnanensis*, together with discrete isolated local clusters, suggests an ancient landscape that imposed little constraint on migration, but which was subsequently disrupted due to regional geo-movements. The discrete differentiation observed at the peripheries appears to reflect both geographic isolation and environment (see the following section).

### Ecological Patterns of Divergence

Three distinct ecotypic clusters (Py-eco1, Py-eco2 and Py-eco3) were identified in *P. yunnanensis*. This division is broadly congruent with that based on mtDNA SAMOVA. The two periphery groups I and VII each corresponded to Py-eco1 and Py-eco3, respectively, and all the other five groups (II–VI) in the central area belonged to Py-eco2. Multivariate analysis showed that environmental elements associated with availability of heat energy and water were the main factors that differentiated the three ecotypic clusters. Heat and water availability have strong impacts on the natural distribution of plant species, and are major determinants of plant productivity [70], [71]. Thus, the niche diversity detected in *P. yunnanensis* could have important consequences for local adaptation and represent an impediment to gene flow. The Py-eco1 region has a humid subtropical climate. It is warmer and wetter than those where the other ecotypes occur, and is similar to the Mio-Pliocene paleo-climate of SW China [72], [73]. Fossil records in SW China indicate that the ancestor of *P. yunnanensis* was present in a milder and moister climate during the Late Miocene than that of today [74]. Triggered by the uplift of the Tibetan Plateau and global cooling in the Late Neogene, *P. yunnanensis* adapted to drier climate in its central distribution area, while in the western periphery (Py-eco1 region) it survived in a warmer and more humid region [74]. Thus, Py-eco1 probably represents a relic ecotype of *P. yunnanensis*. The Py-eco3 region represents a much drier climate than Py-eco1. Populations in this region have a distinct morphology characterized by thin and pendulous needles [35], which is considered to be an adaptation to dry and hot environments [75] [76]. In addition, it has been suggested that a foehn wind specific to the Py-eco3 region is critical for pollination and cone splitting in local *P. yunnanensis* populations [75]. Based on their morphological divergence, some authors [35], [77] classified the populations in this region as a variety or ecotype of *P. yunnanensis*. The congruence between genetic and ecological divisions suggests that environmental adaptation could have contributed to the genetic divergence of groups I and VII. Py-eco2 covers the major range of the species, including population groups II–VI. In this region/environment, population differentiation is characterized by IBD as shown by a partial Mantel test. Thus, the genetic groups recognized in this region were shaped by historically neutral processes and local barriers to gene flow rather than by ecological selection.

Neutral DNA markers are not expected to reflect the history of natural selection and adaptation. However, maternally inherited mtDNA, which is dispersed through seeds, is often used to track population establishment and migration history [39], [78], [79]. Population establishment and forest regeneration is brought about via seeds. Local adaptation to a distinct niche would result in ecological selection against immigrants [3]. Given enough time since a population began to diverge, drift and selection could induce fixation of distinct mitotypes in different niches, and thus shape the genetic structure of local populations [3], [8], [78]. In this context, the mtDNA pattern might indirectly reflect a population’s persistence in, and adaptation to, a specific niche. Most boreal and temperate forest trees retreated into refugia during the Last Glacial Maximum and their current distribution ranges are the result of post-glaciation colonization [80], [81], [82]. In these species, adaptive evolution may have had insufficient time to induce distinct genetic divergence in recently colonized regions, and population structures revealed by neutral markers have been shaped mainly by periodical isolation and range expansion. In contrast, *P. yunnanensis* is distributed in a subtropical region that is recognized as having been a refuge during the last glaciations [17], [83], where population demography was less influenced by climate fluctuations. Therefore, ecological divergence could have developed into a barrier preventing immigrants from surviving and reproducing in new habitats [3], and further strengthened genetic differentiation between *P. yunnanensis* niches. The impact of immigrant inferiority (or inviability) is less visible in a genome that is dispersed through pollen (cpDNA in pines) than in the seeds that produce immigrant plant individuals [3], [84]. Immigrant inviability commonly exists between populations that exhibit adaptive ecological divergence, and it plays an important role in ecological modes of speciation [3]. Our finding that ecological and geographic distances have had a significant effect on species-wide genetic divergence supports the hypothesis that both environment and geographic factors contributed to genetic differentiation in *P. yunnanensis*. The selective pressure exerted by niche divergence upon fitness in this species remains to be explicitly tested.

### Conclusions

Integrating the results of genetic analysis and ecological niche modeling revealed the occurrence of ecological and phylogeographic processes in *P. yunnanensis* that were different from those seen in other case studies in SW China. In other taxa from the same region, the intraspecific genetic structure reflects the major role played by historic drainage systems. In contrast, in the case of *P. yunnanensis* our observation of continuous genetic differentiation over the majority of its range, together with discrete isolated local clusters, suggests a paleo-landscape that was generally well connected and imposed few migration constraints, but which was subsequently disrupted as a result of geomorphological movements in response to the uplift of the eastern Tibetan Plateau. The finding of discrete differentiation between two genetic groups in the peripheries is consistent with niche divergence and geographical isolation of these groups. In the central area of the species’ range, population structure was shaped mainly by neutral processes and local geography rather than by ecological selection. These results show that geographical and environmental factors acting in combination have created stronger and more discrete genetic differentiation than IBD alone, and illustrate the importance of ecological factors in promoting and maintaining genetic divergence across a complex landscape. Our study highlights the heterogeneous contributions made by historic neutral processes and environment to genetic variation among different taxa in SW China. Further research incorporating multiple approaches applied to additional taxa would permit a better understanding of the origin and maintenance of biological diversity in this geologically unique region.

## Supporting Information

Figure S1 **SAMOVA analysis of mtDNA and cpDNA. X-axis shows different** ***K*** **values (number of groups) and Y-axis shows corresponding** ***F*** **_CT_ values.**
(TIF)

Table S1 **148 occurrence records for** ***Pinus yunnanensis*** **, with the corresponding 14 environmental variables.** The eight variables used in ecological niche modeling are indicated in bold.
(PDF)

Table S2 **The frequencies of mitotypes and chlorotypes in the 16 sampled** ***Pinus yunnanensis*** **populations.**
(PDF)
