# Supplementary figures and images for: Impact of Geography and Climate on the Genetic Differentiation of the Subtropical Pine Pinus yunnanensis
Source: PLoS One. 2013 Jun 26;8(6):e67345. doi: 10.1371/journal.pone.0067345 (PMC3693954; doi:10.1371/journal.pone.0067345)

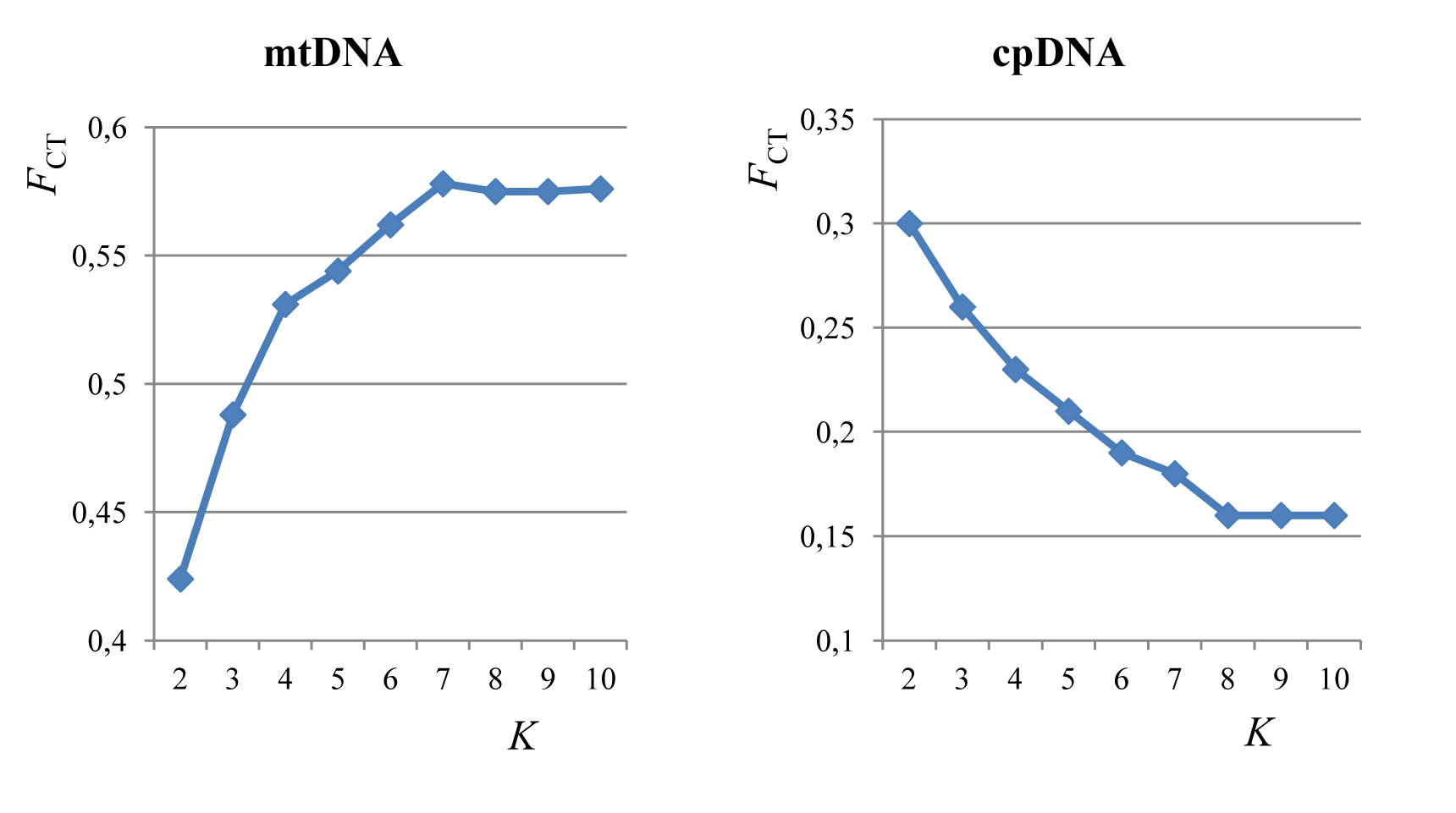

Supplement: Figure S1 — SAMOVA analysis of mtDNA and cpDNA. X-axis shows different K values (number of groups) and Y-axis shows corresponding F CT values. (TIF) [file pone.0067345.s001.tif]
