# Supplementary material for: Impact of Geography and Climate on the Genetic Differentiation of the Subtropical Pine Pinus yunnanensis
Source: PLoS One. 2013 Jun 26;8(6):e67345. doi: 10.1371/journal.pone.0067345 (PMC3693954; doi:10.1371/journal.pone.0067345)
Supplement: Table S1 — 148 occurrence records for Pinus yunnanensis , with the corresponding 14 environmental variables. The eight variables used in ecological niche modeling are indicated in bold. (PDF) [file pone.0067345.s002.pdf]

Table S1. 148 occurrence records for *Pinus yunnanensis* , with the corresponding 14 environmental variables. The eight variables used in ecological niche modeling are indicated in bold.

					Environmental variables													
No.	Longitude (E)	Latitude (N)	Altitude (m)	TwoStep	bio1	bio2	**bio3**	**bio4**	**bio5**	**bio12**	bio14	**bio15**	**wet**	vap	**sph**	**sc**	gdd	frs
1	98.3	25.31667	1782	1	149	112	48	4292	231	1573	16	77	17.02000046	14.47999954	5.620999813	6.28000021	3110.600098	4.010000229
2	98.35	25.1	1635	1	158	113	48	4265	242	1562	18	78	17.37000084	14.17000008	5.785999775	5.324999809	3262.419922	4.099999905
3	98.46133	25.32508	1908	1	150	112	48	4325	233	1483	17	77	17.86000061	13.35000038	5.952000141	5.471000195	3026.189941	4.739999771
4	98.65	25.03333	1629	1	160	114	48	4361	245	1372	19	76	17.86000061	14.05000019	6.40899992	4.149000168	3384.030029	4.059999943
5	98.68333	25.16667	1627	1	160	114	48	4394	246	1351	19	74	17.93000031	13.56999969	6.422999859	4.183000088	3127.469971	4.440000057
6	98.75	25.85	2597	1	137	111	48	4342	222	1363	13	81	17.30999947	11.85999966	6.56099987	4.842000008	2199.800049	5.789999962
7	98.76667	24.95	2089	1	140	114	49	4315	225	1277	16	78	17.72999954	14.34000015	6.611999989	3.848999977	3569.5	3.75999999
8	98.78333	24.63333	1828	1	151	116	49	4221	238	1314	16	80	17.22999954	14.64999962	6.418000221	3.954999924	3931.129883	3.400000095
9	98.8	25.16667	1864	1	165	114	48	4427	251	1266	18	73	17.90999985	13.60999966	6.607999802	3.834000111	3182.300049	4.360000134
10	98.82419	25.96578	1503	1	175	110	47	4386	261	1473	19	69	17.04999924	11.35999966	6.629000187	4.915999889	2030.050049	6.170000076
11	98.83333	26.15	1705	1	168	110	47	4461	255	1479	17	72	16.65999985	10.59000015	6.563000202	5.276000023	1746.5	6.900000095
12	98.85	24.58333	2025	1	146	115	49	4227	232	1287	15	80	17.28000069	14.52000046	6.285999775	3.937000036	3941.469971	3.450000048
13	98.87247	26.20326	1316	1	185	109	47	4418	273	1563	19	66	16.54000092	10.05000019	6.606999874	5.229000092	1670.27002	7.380000114
14	99.01667	25.86667	1530	1	174	112	48	4407	262	1318	19	68	17.23999977	10.72999954	7.021999836	4.175000191	2212.669922	6.590000153
15	99.067	25.684	2199	1	142	112	48	4381	230	1213	15	77	17.5	11.48999977	7.140999794	3.884000063	2528.070068	5.929999828
16	99.08333	24.86667	1673	1	161	115	48	4392	250	1110	16	75	17.57999992	14.40999985	6.401000023	3.734999895	3754.629883	3.539999962
17	99.13807	24.47679	1926	1	150	115	49	4217	238	1212	15	81	17.15999985	14.60999966	6.085999966	3.703999996	4121.450195	3.200000048
18	99.2	26.73333	2752	1	104	109	47	4511	196	1109	8	88	15.47000027	6.840000153	6.797999859	3.895999908	1109	10.34000015
19	99.2029	24.7191	1647	1	163	116	49	4323	253	1118	16	77	17.27000046	14.51000023	6.070000172	3.663000107	3834.320068	3.319999933
20	99.25	24.61667	1818	1	152	115	49	4270	241	1138	15	80	17.09000015	14.71000004	6.038000107	3.612999916	3974.25	3.099999905
21	99.25	25.999	2926	1	111	110	48	4345	198	1167	10	86	16.70000076	10.18999958	7.352000237	3.588999987	2088.5	6.809999943
22	99.32004	26.59541	2875	1	107	109	47	4470	199	1106	8	88	15.56999969	7.380000114	6.872000217	3.727999926	1272.27002	9.619999886
23	99.35	25.81667	1779	1	170	112	48	4441	261	1144	17	69	16.76000023	11.22999954	7.388999939	3.602999926	2286.189941	5.920000076
24	99.35688	26.4795	2546	1	127	110	48	4458	218	1145	10	85	15.68999958	7.940000057	6.923999786	3.769000053	1430.599976	8.970000267
25	99.4	26.16667	2145	1	148	111	48	4407	238	1156	13	78	16.20000076	9.340000153	7.116000175	3.812999964	1843.339966	7.5
26	99.42936	25.89479	2107	1	148	111	48	4405	237	1127	14	78	16.56999969	10.80000019	7.288000107	3.703000069	2167.090088	6.190000057
27	99.55	26.7	2601	1	115	111	48	4489	209	1064	7	91	15.28999996	6.820000172	6.478000164	3.894999981	1115.900024	10.06999969
28	99.6	26	2508	1	128	111	49	4353	218	1100	11	86	16.20999908	9.899999619	7.084000111	3.915999889	2009.400024	6.760000229
29	99.67116	25.40889	2423	1	130	111	49	4265	218	1080	14	82	16.47999954	13.5	7.210000038	3.846999884	3261.219971	3.829999924

Continued Table S1

					Environmental variables													
No.	Longitude (E)	Latitude (N)	Altitude (m)	TwoStep	bio1	bio2	**bio3**	**bio4**	**bio5**	**bio12**	bio14	**bio15**	**wet**	vap	**sph**	**sc**	gdd	frs
30	99.76667	26.1	1889	1	160	112	**48**	**4409**	**253**	**1065**	14	**77**	**15.76000023**	9.199999809	**6.868999958**	**4.131999969**	1944.76001	7.300000191
31	99.85	26.3	2141	1	147	112	**48**	**4381**	**240**	**1049**	10	**84**	**15.32999992**	8.399999619	**6.665999889**	**4.212999821**	2016.699951	8.050000191
32	99.93333	25.98333	2237	1	142	111	**48**	**4335**	**235**	**1053**	12	**85**	**15.63000011**	9.399999619	**6.876999855**	**4.152999878**	2512.120117	6.960000038
33	99.95	26.86667	1864	1	165	115	**48**	**4519**	**262**	**1012**	10	**79**	**14.85999966**	5.489999771	**6.217999935**	**3.881999969**	1165.699951	11.34000015
34	99.96667	24.83333	2297	1	137	111	**49**	**4112**	**225**	**1080**	14	**83**	**16.52000046**	12.72999954	**6.428999901**	**3.892999887**	3830.639893	4.139999866
35	99.96667	25.08333	1500	1	180	114	**48**	**4271**	**275**	**986**	15	**76**	**16.32999992**	12.85999966	**6.81099987**	**3.812000036**	3813.330078	4.019999981
36	100.0333	25.7	2142	1	147	111	**48**	**4281**	**242**	**1047**	14	**83**	**15.59000015**	11.60999966	**6.940999985**	**4.138000011**	2966.300049	4.920000076
37	100.0833	25.98333	2136	1	148	112	**48**	**4323**	**243**	**1034**	12	**85**	**15.31000042**	9.640000343	**6.78399992**	**4.244999886**	2960	6.539999962
38	100.131	25.68548	2608	1	123	111	**49**	**4235**	**214**	**1058**	11	**89**	**15.39000034**	11.82999992	**6.848999977**	**4.21999979**	3203.300049	4.599999905
39	100.1668	25.25421	2357	1	135	111	**48**	**4189**	**227**	**1049**	13	**86**	**15.67000008**	13.13000011	**6.953000069**	**3.654000044**	3811.090088	3.549999952
40	100.1833	25.1667	2206	1	138	111	**49**	**4155**	**229**	**1047**	13	**85**	**15.72999954**	13.17000008	**6.794000149**	**3.72300005**	3868.620117	3.539999962
41	100.1833	26.15	2059	1	153	113	**49**	**4341**	**249**	**1008**	11	**85**	**14.81000042**	9.720000267	**6.679999828**	**4.27699995**	3266.909912	6.349999905
42	100.2	25.01667	1976	1	165	112	**48**	**4171**	**259**	**997**	15	**81**	**15.80000019**	13.28999996	**6.472000122**	**3.903000116**	3979.629883	3.480000019
43	100.3034	25.22222	1710	1	172	113	**48**	**4215**	**268**	**972**	14	**80**	**15.38000011**	13.31000042	**6.734000206**	**3.723999977**	3857.679932	3.289999962
44	99.32483	27.19582	2426	2	129	111	**47**	**4659**	**226**	**1100**	8	**85**	**14.63000011**	6.110000134	**6.355000019**	**5.300000191**	1336.98999	11.18999958
45	99.43	26.95	2893	2	106	110	**47**	**4596**	**202**	**1043**	7	**90**	**15.13000011**	5.619999886	**6.468999863**	**4.276000023**	1178.890015	11.56999969
46	99.46607	27.6	2471	2	126	114	**47**	**4789**	**228**	**991**	7	**87**	**13.81000042**	6.5	**6.340000153**	**5.190000057**	1259.150024	10.89000034
47	99.46667	27.2	2507	2	125	112	**47**	**4638**	**223**	**1043**	7	**88**	**14.63000011**	5.71999979	**6.281000137**	**4.721000195**	1329.310059	11.52999973
48	99.509	28.015	3225	2	78	140	**48**	**5185**	**195**	**906**	3	**96**	**13.53999996**	4.320000172	**6.225999832**	**5.913000107**	932.2700195	14.02000046
49	99.5764	28.02422	3320	2	78	140	**48**	**5181**	**194**	**904**	3	**96**	**13.53999996**	4.320000172	**6.225999832**	**5.913000107**	932.2700195	14.02000046
50	99.65	27.1333	2312	2	139	113	**47**	**4587**	**236**	**1039**	7	**88**	**14.71000004**	5.340000153	**6.204999924**	**4.004000187**	1277.680054	11.81000042
51	99.7167	26.7833	2720	2	114	112	**48**	**4490**	**209**	**1023**	6	**94**	**15.06999969**	6.21999979	**6.18900013**	**3.983999968**	1049.619995	10.64000034
52	99.8333	26.8667	2689	2	116	113	**48**	**4508**	**213**	**1002**	5	**96**	**14.94999981**	5.599999905	**6.175000191**	**3.903000116**	1128.069946	11.28999996
53	99.85	26.65	2610	2	126	113	**48**	**4422**	**221**	**1025**	6	**94**	**14.98999977**	6.880000114	**6.302999973**	**4.048999786**	1302	9.760000229
54	99.91667	26.43333	2371	2	135	112	**48**	**4403**	**229**	**1035**	7	**91**	**15.03999996**	7.900000095	**6.53000021**	**4.147999763**	1912.030029	8.510000229
55	99.91715	26.538	2198	2	145	113	**48**	**4431**	**240**	**1028**	8	**88**	**14.93000031**	7.460000038	**6.427000046**	**4.092000008**	1650.079956	9.010000229
56	99.95536	26.76798	2650	2	120	113	**48**	**4447**	**216**	**1002**	5	**97**	**14.86999989**	6.070000172	**6.235000134**	**3.959000111**	1169.619995	10.64000034
57	100.05	27.18	2202	2	135	115	**48**	**4584**	**233**	**996**	6	**91**	**14.60000038**	5.21999979	**6.15199995**	**3.671000004**	1250.680054	11.85999966
58	100.2	26.5	2203	2	145	114	**49**	**4402**	**241**	**993**	7	**93**	**14.18999958**	9.260000229	**6.500999928**	**4.090000153**	2356.600098	6.860000134
59	100.2167	26.36667	1975	2	157	114	**48**	**4374**	**254**	**988**	9	**86**	**14.34000015**	9.590000153	**6.574999809**	**4.175000191**	2905.98999	6.46999979

Continued Table S1

No.	Longitude (E)	Latitude (N)	Altitude (m)	TwoStep	Environmental variables													
					bio1	bio2	**bio3**	**bio4**	**bio5**	**bio12**	bio14	**bio15**	**wet**	vap	**sph**	**sc**	gdd	frs
60	100.2364	26.88652	2508	2	126	116	49	4468	225	972	3	102	14.31000042	6.21999979	6.30700016	3.823999882	1234.199951	10.39000034
61	100.2667	26.8	2395	2	134	116	49	4459	234	975	3	101	14.18000031	7.159999847	6.330999851	3.915999889	1359.51001	9.300000191
62	100.3633	25.97407	2932	2	102	112	49	4246	194	1052	8	96	14.60000038	11.02999973	6.602000237	4.46999979	3562.540039	4.809999943
63	100.4	25.96	1649	2	175	115	48	4308	275	955	12	80	14.38000011	11.09000015	6.599999905	4.453999996	3606.199951	4.670000076
64	100.4729	24.89589	2041	2	155	112	49	4091	247	1018	13	86	15.09000015	14.14999962	5.989999771	4.139999866	4083.689941	2.549999952
65	100.5	24.8	1960	2	167	113	49	4068	260	1003	14	84	15.01000023	14.35999966	5.853000164	4.160999775	4136.5	2.359999895
66	100.5	27.2	1683	2	149	119	48	4586	250	934	4	97	14.05000019	4.96999979	6.221000195	4.131999969	1314.150024	11.88000011
67	100.53	25.2	2220	2	144	112	49	4123	238	1020	13	87	14.89000034	13.60000038	6.247000217	4.144999981	3907.929932	2.880000114
68	100.5867	25.22014	1841	2	161	113	49	4136	257	978	13	85	14.81000042	13.51000023	6.15199995	4.254000187	3903.469971	2.950000048
69	100.6	26.6	2419	2	122	116	49	4381	220	993	4	102	13.19999981	10.65999985	6.238999844	4.625999928	2446.77002	5.21999979
70	100.6333	27.16667	2332	2	137	120	49	4544	238	944	3	101	13.72000027	5.659999847	6.224999905	4.402999878	1538.189941	10.97000027
71	100.7	25.16	1815	2	164	113	48	4117	260	963	13	86	14.69999981	13.57999992	5.894000053	4.421000004	3944.77002	2.920000076
72	100.7667	25.7	1952	2	161	115	48	4260	260	950	11	88	14.15999985	12.51000023	5.962999821	5.236999989	3837.850098	3.5
73	100.7833	26.13333	1922	2	162	117	49	4330	262	945	9	91	13.51000023	11.93999958	6.559000015	4.585000038	3828.02002	3.769999981
74	100.7845	27.7098	2692	2	117	127	49	4755	222	900	3	101	13.59000015	4.510000229	6.605000019	4.703000069	1233.109985	12.65999985
75	100.7962	27.2971	2524	2	124	122	49	4603	226	945	3	103	13.35999966	5.960000038	6.315999985	4.633999825	1665.449951	10.60000038
76	100.8004	25.5118	2076	2	156	114	49	4193	253	962	11	89	14.34000015	12.84000015	5.869999886	4.980000019	3841.699951	3.349999905
77	100.8333	25.4	2167	2	149	113	48	4164	245	984	11	89	14.39000034	13.06000042	5.802999973	4.827000141	3845.120117	3.230000019
78	100.8333	26.53333	2276	2	142	118	48	4401	243	974	5	99	12.77000046	11.61999989	6.263999939	4.809999943	2929.47998	4.130000114
79	100.9	25.15	1801	2	173	114	48	4136	269	929	12	86	14.47999954	13.47999954	5.664999962	4.55700016	3877	3.00999999
80	100.9	25.33333	2114	2	153	114	48	4176	250	973	11	89	14.34000015	13.15999985	5.705999851	4.763999939	3807.419922	3.180000067
81	100.9	25.7	2610	2	126	114	49	4173	221	1024	9	95	13.94999981	12.57999992	5.874000072	5.206999779	3719.050049	3.440000057
82	101.0167	26.41667	1849	2	168	120	48	4432	272	927	7	93	12.64000034	12.01000023	6.407999992	4.690000057	3217.189941	3.720000029
83	101.16667	27.33333	2695	2	112	124	49	4625	214	979	3	103	12.63000011	7.539999962	6.267000198	4.986000061	2029.22998	8.630000114
84	101.2631	25.15278	1878	2	170	115	48	4172	268	914	11	88	14.14000034	13.27999973	5.375	4.732999802	3685.23999	3.339999914
85	101.2789	26.65936	1493	2	187	122	48	4573	294	901	7	92	12.52999973	10.5	6.102000237	5.136000156	3494.280029	5.260000229
86	101.37	25.52361	2205	2	152	116	48	4200	250	949	9	93	13.81000042	12.35000038	5.636000156	4.863999844	3633.929932	4.050000191
87	101.5	29	3336	2	44	115	44	5205	154	744	6	93	13.31999969	6.03000021	6.480999947	5.360000134	941.4000244	11.72000027
88	101.509	29.00745	3129	2	44	115	44	5205	154	744	6	93	13.31999969	6.03000021	6.480999947	5.360000134	941.4000244	11.72000027
89	101.6	24.83333	1999	2	165	112	48	4098	259	953	12	88	14.22000027	13.69999981	6.124000072	4.164000034	3848.25	3.170000076

Continued Table S1

					Environmental variables													
No.	Longitude (E)	Latitude (N)	Altitude (m)	TwoStep	bio1	bio2	**bio3**	**bio4**	**bio5**	**bio12**	bio14	**bio15**	**wet**	vap	**sph**	**sc**	gdd	frs
90	101.66	25.12833	1792	2	175	115	**48**	**4167**	**273**	**879**	10	**89**	**13.84000015**	13.14999962	**6.369999886**	**4.195000172**	3645.449951	3.630000114
91	101.85	25.65	1153	2	216	126	**49**	**4433**	**324**	**670**	5	**92**	**13.25**	12.35000038	**5.931000233**	**4.446000099**	4103.600098	4.039999962
92	101.86667	27.5	2433	2	135	121	**48**	**4678**	**237**	**1036**	5	**99**	**12.23999977**	8.600000381	**6.217000008**	**4.761000156**	2374.949951	7.369999886
93	101.9122	25.19083	1750	2	176	115	**48**	**4226**	**274**	**866**	9	**90**	**13.63000011**	12.86999989	**6.714000225**	**4.124000072**	3538.780029	3.900000095
94	102.015	26.93038	2060	2	148	122	**47**	**4778**	**255**	**1073**	6	**101**	**11.5**	12.27999973	**5.925000191**	**4.672999859**	3204.48999	3.710000038
95	102.033	24.13073	1595	2	188	110	**48**	**4055**	**282**	**984**	13	**83**	**14.56999969**	15.85000038	**6.103000164**	**3.890000105**	4413.899902	1.429999948
96	102.1018	27.87644	2118	2	148	116	**46**	**4777**	**252**	**1024**	5	**95**	**12.5**	7.980000019	**6.407999992**	**4.096000195**	2505.689941	8.279999733
97	102.1589	24.25606	1805	2	176	109	**48**	**4070**	**271**	**990**	13	**84**	**14.26000023**	15.31999969	**5.974999905**	**4.157000065**	4198.109863	1.75
98	102.2114	24.20824	1343	2	203	111	**48**	**4141**	**300**	**901**	11	**81**	**14.28999996**	15.32999992	**5.979000092**	**4.146999836**	4191.839844	1.730000019
99	102.2333	24.83333	1706	2	180	111	**48**	**4281**	**274**	**895**	11	**86**	**13.65999985**	13.53999996	**6.552999973**	**4.026000023**	3558.550049	3.25
100	102.2639	24.97083	1851	2	167	109	**47**	**4204**	**261**	**926**	11	**88**	**13.56000042**	13.02000046	**6.679999828**	**4.102000237**	3487.580078	3.710000038
101	102.2939	25.30611	1846	2	164	112	**47**	**4273**	**260**	**908**	10	**91**	**13.36999989**	12.31999969	**6.856999874**	**4.249000072**	3312.070068	4.21999979
102	102.3	24.73	2441	2	132	105	**47**	**4138**	**221**	**1073**	13	**89**	**13.68000031**	13.76000023	**6.410999775**	**4.011000156**	3573.429932	3
103	102.3053	24.37861	2379	2	140	105	**48**	**4007**	**228**	**1101**	15	**88**	**13.97999954**	14.77000046	**6.070000172**	**4.224999905**	3941.48999	2.099999905
104	102.3167	24.5167	2382	2	137	105	**48**	**4092**	**225**	**1083**	14	**88**	**13.80000019**	14.39999962	**6.203000069**	**4.149000168**	3782.409912	2.380000114
105	102.4102	24.83994	1938	2	161	107	**47**	**4204**	**252**	**969**	12	**88**	**13.56000042**	13.25	**6.386000156**	**4.09499979**	3470.199951	3.410000086
106	102.42121	27.59382	2120	2	144	115	**46**	**4833**	**249**	**1026**	6	**95**	**12.47000027**	8.529999733	**5.965000153**	**4.089000225**	2375.159912	7.599999905
107	102.4828	23.8125	1401	2	198	110	**49**	**4010**	**292**	**1004**	11	**84**	**14.57999992**	15.40999985	**6.046000004**	**3.674999952**	4285.540039	1.519999981
108	102.4968	25.05	2269	2	133	106	**47**	**4221**	**223**	**1040**	12	**90**	**13.40999985**	12.56999969	**6.376999855**	**4.277999878**	3342.929932	3.970000029
109	102.5333	24.53333	2200	2	146	104	**47**	**4148**	**235**	**1049**	14	**87**	**13.67000008**	13.97000027	**6.138999939**	**4.28000021**	3648.810059	2.650000095
110	102.585	24.80694	1953	2	161	105	**47**	**4252**	**251**	**986**	12	**86**	**13.51000023**	13.11999989	**6.18599987**	**4.138999939**	3404.77002	3.440000057
111	102.62	25.86417	2156	2	141	115	**47**	**4498**	**239**	**952**	8	**95**	**13.15999985**	10.81999969	**6.073999882**	**4.093999863**	2926.409912	5.380000114
112	102.6226	24.96881	2185	2	141	104	**46**	**4251**	**231**	**1028**	13	**89**	**13.42000008**	12.64000034	**6.169000149**	**4.289999962**	3314.679932	3.890000105
113	102.6263	25.89998	2286	2	132	114	**47**	**4487**	**230**	**982**	8	**96**	**13.15999985**	10.73999977	**6.03000021**	**4.117000103**	2916.050049	5.440000057
114	102.8185	25.29437	2080	2	146	106	**46**	**4388**	**239**	**986**	12	**89**	**13.35000038**	11.89999962	**5.864999771**	**4.604000092**	3123.840088	4.429999828
115	102.8389	25.19472	1977	2	150	105	**46**	**4380**	**242**	**993**	12	**88**	**13.35000038**	12.06000042	**5.860000134**	**4.638999939**	3177.050049	4.309999943
116	102.892	24.92323	2057	2	151	103	**45**	**4395**	**243**	**1022**	13	**87**	**13.38000011**	12.53999996	**5.90199995**	**4.455999851**	3355.840088	3.910000086
117	102.892	24.99617	2071	2	146	103	**45**	**4341**	**236**	**1026**	13	**88**	**13.34000015**	12.38000011	**5.875999928**	**4.518000126**	3313.439941	4.059999943
118	102.9053	24.76194	2705	2	113	101	**46**	**4175**	**200**	**1139**	13	**89**	**13.44999981**	12.85999966	**5.952000141**	**4.333000183**	3450.98999	3.599999905
119	102.9479	25.32931	2157	2	140	106	**45**	**4425**	**233**	**995**	11	**89**	**13.38000011**	11.73999977	**5.743000031**	**4.656000137**	3146.379883	4.53000021

Continued Table S1

					Environmental variables													
No.	Longitude (E)	Latitude (N)	Altitude (m)	TwoStep	bio1	bio2	**bio3**	**bio4**	**bio5**	**bio12**	bio14	**bio15**	**wet**	vap	**sph**	**sc**	gdd	frs
120	102.95	23.88333333	2062	2	156	102	47	4105	247	1100	13	85	13.81999969	14.84000015	5.842000008	4.000999928	4010.409912	1.75
121	102.9825	24.44278	1682	2	180	107	47	4325	275	951	11	84	13.56000042	13.40999985	5.93900013	4.362999916	3771.030029	3.049999952
122	102.9975	25.1625	2029	2	147	104	45	4417	240	1002	12	88	13.35000038	12	5.731999874	4.739999771	3261.399902	4.329999924
123	103.0181	24.26404	2475	2	128	100	46	4139	216	1151	14	87	13.60000038	13.81999969	5.921000004	4.256999969	3970.330078	2.630000114
124	103.1766	24.73217	1838	2	161	105	45	4429	256	1002	13	85	13.28999996	13.18000031	5.827000141	4.611000061	3666.679932	3.279999971
125	103.2016	24.97649	1564	2	177	108	46	4532	273	961	11	84	13.28999996	12.65999985	5.668000221	4.802999973	3489.280029	3.74000001
126	103.2467	25.42306	1900	2	157	108	45	4566	253	927	11	86	13.47999954	11.75	5.43200016	4.768000126	3159.48999	4.400000095
127	103.305	24.52444	1955	2	155	105	46	4376	249	1014	13	84	13.13000011	13.93999958	5.797999859	4.339000225	3894.600098	2.599999905
128	103.45984	24.16696	1552	2	175	106	46	4382	272	944	11	82	13.34000015	14.09000015	5.919000149	4.197000027	4032.040039	2.279999971
129	103.50151	24.21653	1733	2	164	105	46	4397	260	979	12	82	13.26000023	14.14000034	5.953000069	4.30700016	4014.830078	2.25
130	103.5167	25.48333	2000	2	152	106	44	4584	247	936	11	84	13.61999989	11.81999969	5.605999947	5.146999836	3208.459961	4.21999979
131	103.8333	25.71667	1936	2	155	104	44	4698	249	933	12	84	14.13000011	11.53999996	5.602000237	5	3230.550049	4.329999924
132	103.9333	25.83333	2078	2	144	102	43	4752	237	972	11	84	14.27999973	11.56000042	5.590000153	4.776000023	3060.820068	4.239999771
133	103.9861	25.71993	2149	2	140	101	43	4719	231	1011	12	83	14.28999996	11.67000008	5.497000217	4.822999954	3070.310059	4.150000095
134	104.0964	23.42715	1418	3	173	87	42	4432	263	1082	12	78	14.85000038	14.69999981	5.78399992	4.066999912	4007.73999	1.649999976
135	104.13773	24.07135	1490	3	175	101	44	4638	270	972	13	76	13.78999996	14.22000027	5.81099987	3.880000114	4013.790039	2.230000019
136	104.35	24.06667	1495	3	171	99	43	4730	266	1009	14	76	13.97000027	14.35000038	5.730000019	3.676000118	4070.290039	2.170000076
137	104.4	24.28333	1757	3	157	99	43	4754	252	1070	16	77	13.77999973	14.5	5.758999825	3.742000103	4101.160156	2.200000048
138	104.49344	23.69962	1536	3	165	91	41	4611	258	1094	14	78	14.56999969	14.67000008	5.69299984	3.757999897	4077.310059	1.649999976
139	104.6649	23.3148	1148	3	184	85	40	4600	279	1255	12	82	14.47999954	16.32999992	5.801000118	4.565000057	4470.120117	0.779999971
140	104.8164	23.19861	1310	3	173	82	39	4596	266	1365	11	86	14.19999981	17.30999947	5.801000118	4.675000191	4743.799805	0.460000008
141	105.07669	24.0749	1270	3	175	100	41	5010	277	1101	14	78	14.14999962	15.10999966	5.829999924	3.595000029	4240.140137	1.870000005
142	105.1875	23.98139	1275	3	175	96	40	5008	276	1149	14	79	14.21000004	15.18999958	5.888999939	3.624000072	4237.350098	1.809999943
143	105.8166667	23.78333333	864	3	193	88	38	5098	295	1268	12	84	13.67000008	16.86000061	6.114999771	3.694999933	4719.379883	0.99000001
144	105.95	23.93333333	762	3	196	89	38	5169	301	1219	10	84	13.52000046	16.89999962	6.080999851	3.676000118	4856.259766	1.019999981
145	106.15	24.7	508	3	219	94	37	5635	335	1137	9	80	13.35000038	16.84000015	5.755000114	3.601000071	4555.560059	1.190000057
146	106.3	24.93333333	587	3	199	92	36	5718	315	1197	10	79	13.59000015	16.56999969	5.71600008	3.61500001	4601.790039	1.389999986
147	106.3166667	24.81666667	908	3	186	90	36	5672	298	1236	10	80	13.46000004	16.77000046	5.697999954	3.572999954	4573.870117	1.279999971
148	106.5667	24.8	1174	3	170	86	35	5632	279	1311	14	78	13.39999962	17.02000046	5.649000168	3.628000021	4601.149902	1.210000038
