# Supplementary material for: Impact of Geography and Climate on the Genetic Differentiation of the Subtropical Pine Pinus yunnanensis
Source: PLoS One. 2013 Jun 26;8(6):e67345. doi: 10.1371/journal.pone.0067345 (PMC3693954; doi:10.1371/journal.pone.0067345)
Supplement: Table S2 — The frequencies of mitotypes and chlorotypes in the 16 sampled Pinus yunnanensis populations. (PDF) [file pone.0067345.s003.pdf]

Table S2. The frequencies of mitotypes and chlorotypes in the 16 sampled *Pinus yunnanensis* populations.

Population		Longitude (E)	Latitude (N)	Altitude (m)	mtDNA*															
					N	n_h	H_e	M17	M18	M19	M20	M21	M22	M23	M24	M25	M26	M27	M28	M29
1	Gongshan	98°49'	25°58'	1616	16	2	0.125	0	0	1	0	0	0	0	15	0	0	0	0	0
2	Tengchong	98°39'	25°02'	1580	16	1	0	0	0	0	0	0	0	0	16	0	0	0	0	0
3	Baoshan	99°08'	24°28'	1897	16	2	0.233	0	0	2	0	0	0	0	14	0	0	0	0	0
4	Zhongdian 1	99°32'	28°09'	3048	16	1	0	0	0	0	0	0	0	0	0	16	0	0	0	0
5	Zhongdian 2	100°03'	27°11'	2009	16	3	0.667	0	0	0	0	0	0	0	8	4	0	4	0	0
6	Lijiang	100°13'	26°53'	2493	16	4	0.675	0	0	8	1	0	0	0	5	0	0	0	0	2
7	Binchuan	100°21'	25°58'	3141	16	3	0.700	0	0	4	0	6	0	0	0	0	6	0	0	0
8	Yuxi	102°09'	24°15'	1849	16	7	0.850	0	1	5	0	0	4	2	2	0	1	0	1	0
9	Shiping	102°29'	23°43'	1428	16	3	0.492	0	1	11	0	0	0	4	0	0	0	0	0	0
10	Jianshui	102°57'	23°50'	2084	16	2	0.500	0	0	6	0	0	0	10	0	0	0	0	0	0
11	Jiulong	101°30'	29°00'	3129	16	2	0.458	0	0	11	0	0	0	0	5	0	0	0	0	0
12	Miyi	102°01'	26°55'	2047	15	4	0.467	1	0	11	0	0	0	0	0	0	1	0	2	0
13	Kunming	102°37'	24°58'	2242	16	2	0.325	0	0	13	0	0	0	0	0	0	0	0	3	0
14	Yiliang	103°10'	24°43'	1846	16	2	0.125	1	0	15	0	0	0	0	0	0	0	0	0	0
15	Luoping	104°24'	24°17'	1643	16	2	0.325	0	3	0	0	0	0	13	0	0	0	0	0	0
16	Leye	106°34'	24°48'	1039	16	1	0	0	0	0	0	0	0	16	0	0	0	0	0	0

Continued Table S2

Population		*N*	n_h	H_e	cpDNA†																								
					C24	C25	C26	C27	C28	C29	C30	C31	C32	C33	C34	C35	C36	C37	C40	C41	C42	C48	C54	C55	C56	C61	C67	C93	C94
1	Gongshan	16	9	0.900	0	0	1	0	3	0	4	0	1	0	1	1	0	0	0	0	0	0	0	1	3	0	0	0	0
2	Tengchong	16	3	0.242	0	0	1	0	1		14	0	0	0	0	0	0	0	0	0	0	0	0	0	0	0	0	0	0
3	Baoshan	16	11	0.933	1	2	1	0	0	0	4	0	0	0	0	0	0	0	0	0	0	0	2	1	1	0	0	0	0
4	Zhongdian 1	16	9	0.917	0	0	2	0	1	0	2	0	4	1	0	1	2	0	0	0	0	0	0	0	0	2	0	0	0
5	Zhongdian 2	16	8	0.858	0	0	0	0	1	1	5	0	1	0	0	1	4	0	0	0	2	0	0	0	0	0	0	0	1
6	Lijiang	16	9	0.900	0	0	3	0	0	1	4	0	0	0	1	0	3	0	1	0	0	0	0	0	0	0	0	0	0
7	Binchuan	16	9	0.883	0	0	0	0	1	0	5	0	1	2	0	3	1	0	0	0	0	0	0	0	0	0	0	1	0
8	Yuxi	16	4	0.692	0	0	7	0	0	1	6	0	0	0	0	0	0	2	0	0	0	0	0	0	0	0	0	0	0
9	Shiping	16	5	0.650	1	0	4	0	0	0	9	0	0	0	0	0	1	0	0	0	0	0	0	0	0	0	0	0	0
10	Jianshui	16	5	0.683	0	0	0	0	1		8	1	0	0	0	5	1	0	0	0	0	0	0	0	0	0	0	0	0
11	Jiulong	16	10	0.825	0	0	0	0	1	1	7	1	1	0	0	1	1	0	1	0	0	1	0	0	0	0	0	0	0
12	Miyi	15	8	0.791	0	0	0	1	1	0	7	0	0	0	0	1	2	1	0	0	1	0	0	0	0	0	1	0	0
13	Kunming	16	8	0.700	0	0	1	0	0	1	9	1	1	0	0	0	1	0	0	0	1	0	0	0	0	0	0	0	0
14	Yiliang	16	8	0.850	1	0	2	1	1	2	6	0	0	0	0	0	0	0	0	2	0	0	0	0	1	0	0	0	0
15	Luoping	16	3	0.342	0	0	0	0	2	0	13	0	0	0	0	0	0	0	0	0	0	0	0	0	0	0	0	0	0
16	Leye	16	2	0.325	0	0	3	0	0	0	13	0	0	0	0	0	0	0	0	0	0	0	0	0	0	0	0	0	0

Continued Table S2

Population		cpDNA†														Population code in Wang *et al*. 2011
		C95	C97	C102	C104	C111	C112	C114	C120	C121	C124	C125	C137	C143	C183	
1	Gongshan	0	0	0	0	0	0	0	1	0	0	0	0	0	0	49
2	Tengchong	0	0	0	0	0	0	0	0	0	0	0	0	0	0	New
3	Baoshan	1	1	1	0	0	0	0	0	0	0	0	1	0	0	50
4	Zhongdian 1	0	0	0	1	0	0	0	0	0	0	0	0	0	0	45
5	Zhongdian 2	0	0	0	0	0	0	0	0	0	0	0	0	0	0	46
6	Lijiang	0	0	0	0	1	0	0	0	0	0	1	0	1	0	47
7	Binchuan	0	0	0	0	0	0	0	0	1	1	0	0	0	0	48
8	Yuxi	0	0	0	0	0	0	0	0	0	0	0	0	0	0	54
9	Shiping	0	0	0	0	0	0	0	0	0	0	0	0	0	1	New
10	Jianshui	0	0	0	0	0	0	0	0	0	0	0	0	0	0	New
11	Jiulong	0	0	0	0	0	0	1	0	0	0	0	0	0	0	44
12	Miyi	0	0	0	0	0	0	0	0	0	0	0	0	0	0	51
13	Kunming	0	0	0	0	0	1	0	0	0	0	0	0	0	0	52
14	Yiliang	0	0	0	0	0	0	0	0	0	0	0	0	0	0	53
15	Luoping	0	0	0	0	1	0	0	0	0	0	0	0	0	0	New
16	Leye	0	0	0	0	0	0	0	0	0	0	0	0	0	0	New

* Mitotype nomenclature (e.g. M17) follows that in Wang *et al*. 2011.

†Chlorotype nomenclature (e.g. C24) follows that in Wang *et al*. 2011; C183 is a new chlorotype detected in this study, its composition of the five SSR site (PCP1289/Pt87268/Pt71936/Pt45002/PCP41131) is 111/158/146/165/134, respectively.

Reference: Wang, B., Mao, J.-F., Gao, J., Zhao, W. & Wang, X.-R. (2011) Colonization of the Tibetan Plateau by the homoploid hybrid pine *Pinus densata*. Molecular Ecology, 20, 3796-3811.
